# Hydrogen Sulfide Abrogates Hemoglobin-Lipid Interaction in Atherosclerotic Lesion

**DOI:** 10.1155/2018/3812568

**Published:** 2018-01-21

**Authors:** László Potor, Péter Nagy, Gábor Méhes, Zoltán Hendrik, Viktória Jeney, Dávid Pethő, Anita Vasas, Zoltán Pálinkás, Enikő Balogh, Ágnes Gyetvai, Matthew Whiteman, Roberta Torregrossa, Mark E. Wood, Sándor Olvasztó, Péter Nagy, György Balla, József Balla

**Affiliations:** ^1^HAS-UD Vascular Biology and Myocardial Pathophysiology Research Group, Hungarian Academy of Sciences, Debrecen 4012, Hungary; ^2^Department of Pediatrics, Faculty of Medicine, University of Debrecen, Debrecen 4012, Hungary; ^3^Department of Molecular Immunology and Toxicology, National Institute of Oncology, Budapest 1122, Hungary; ^4^Department of Pathology, Faculty of Medicine, University of Debrecen, Debrecen 4012, Hungary; ^5^Division of Nephrology, Department of Medicine, Faculty of Medicine, University of Debrecen, Debrecen 4012, Hungary; ^6^Department of Inorganic and Analytical Chemistry, University of Debrecen, Debrecen 4032, Hungary; ^7^University of Exeter Medical School, Exeter, UK; ^8^College of Life and Environmental Sciences, University of Exeter, Exeter, UK; ^9^Division of Vascular Surgery, Department of Surgery, Faculty of Medicine, University of Debrecen, Debrecen 4012, Hungary

## Abstract

The infiltration of red blood cells into atheromatous plaques is implicated in atherogenesis. Inside the lesion, hemoglobin (Hb) is oxidized to ferri- and ferrylHb which exhibit prooxidant and proinflammatory activities. Cystathione gamma-lyase- (CSE-) derived H_2_S has been suggested to possess various antiatherogenic actions. Expression of CSE was upregulated predominantly in macrophages, foam cells, and myofibroblasts of human atherosclerotic lesions derived from carotid artery specimens of patients. A similar pattern was observed in aortic lesions of apolipoprotein E-deficient mice on high-fat diet. We identified several triggers for inducing CSE expression in macrophages and vascular smooth muscle cells including heme, ferrylHb, plaque lipids, oxidized low-density lipoprotein, tumor necrosis factor-*α*, and interleukin-1*β*. In the interplay between hemoglobin and atheroma lipids, H_2_S significantly mitigated oxidation of Hb preventing the formation of ferrylHb derivatives, therefore providing a novel function as a heme-redox-intermediate-scavenging antioxidant. By inhibiting Hb-lipid interactions, sulfide lowered oxidized Hb-mediated induction of adhesion molecules in endothelium and disruption of endothelial integrity. Exogenous H_2_S inhibited heme and Hb-mediated lipid oxidation of human atheroma-derived lipid and human complicated lesion. Our study suggests that the CSE/H_2_S system represents an atheroprotective pathway for removing or limiting the formation of oxidized Hb and lipid derivatives in the atherosclerotic plaque.

## 1. Introduction

Atherosclerosis-related morbidity and mortality are closely associated with the presence of vulnerable plaques and complicated lesions [1–3]. These lesions contain products of lipid peroxidation such as lipid hydroperoxides, aldehydes and carbonyls, calcium, and redox active iron [4]. Vascular lesions are called complicated where due to a disruption of the atheromatous plaque, infiltration of red blood cells (RBCs) are visualized inside the lesion [2]. It was recently shown by Michel et al. that the neovascularization could be observed from the adventitia through the media into the plaque [5]. Often, RBCs infiltrate these lesions as a consequence of leaky neovessels or intraplaque hemorrhage [3, 6]. It was shown that part of the damaged RBCs are taken up by macrophages via erythrophagocytosis and degraded in the lysosome. The generated iron could be exocytosed from the macrophages to the extracellular space and induce oxidation of the LDL and its uptake by macrophages [7–9]. It was shown that invading RBCs lyse and release hemoglobin (Hb) inside the plaque and react with the surrounding plaque lipids [10–12]. In the reactions between Hb and plaque lipids, different oxidized Hb derivatives are formed including metHb (Fe^3+^) and ferrylHb (Fe^4+^=O^2−^) species [10–12]. Furthermore, the ferryl form is unstable and triggers an electron transfer from proximal amino acids of the globin chain to iron, resulting in globin radical formation. Termination reactions of globin radicals yield covalently cross-linked Hb multimers which are present in human complicated atherosclerotic lesions [10, 12].

Oxidized Hb species exert different prooxidant and proinflammatory effects. Both metHb and ferrylHb (we herein use the term ferrylHb to refer to the sum of all those oxidized Hb species in which production the unstable Fe^4+^ oxidation state has been involved) sensitize vascular endothelial cells to oxidant-mediated killing [11, 13] and induce lipid peroxidation via the release of heme and redox active iron [11, 14]. Oxidized Hb has been recently shown to provoke the rearrangement of F-actin cytoskeleton and subsequently the formation of intercellular gaps in endothelium *in vitro* and facilitate the adherence of monocytes to the endothelium through the induction of adhesion molecules: vascular cell adhesion molecule-1 (VCAM-1), intercellular adhesion molecule-1 (ICAM-1), and E-selectin [11, 15].

Hydrogen sulfide (H_2_S), recently proposed as the newest member of the gaseous mediators' family, has been shown to possess antiatherogenic effects in different animal models (reviewed in [16]). Wang et al. previously reported that atherosclerotic lesion formation is inhibited by sodium hydrosulfide (NaSH, an inorganic source of sulfide) in apolipoprotein E knockout mice (ApoE^−/−^), whereas pharmacological inhibition of cystathionine gamma-lyase (CSE), a key enzyme of H_2_S generation in the vasculature, resulted in accelerated plaque formation [17]. A more recent and robust study by Mani et al. using CSE knockout mice showed decreased vascular production of H_2_S accompanied by accelerated atherosclerotic plaque progression compared to wild-type animal highlighting the central importance of CSE and H_2_S as endogenous vasoprotective mediators [18].

Based on these observations, we aimed to investigate whether hydrogen sulfide inhibits hemoglobin-lipid interactions in atherosclerotic lesions and alter subsequent endothelial cell reactions. We also determined how atherogenesis influenced the vascular expression of CSE and identified pathophysiological modulators of CSE expression.

## 2. Materials and Methods

### 2.1. Materials

All chemicals were analytical reagent grade or better and purchased from Sigma-Aldrich (St. Louis, MO, USA). The sulfide donor molecules used in this study—GYY4137 (P-(4-methoxyphenyl)-P-4-morpholinylphosphinodithioic acid morpholine salt), AP67 (4-methoxyphenyl)(pyrrolidin-1-yl)phosphinodithioc acid), and AP72 (4-methoxyphenyl)(piperidin-1-yl)phosphinodithioc acid)—were synthesized in-house [19, 20]. Sulfide stock solutions were prepared fresh daily in water and used immediately.

### 2.2. Human Tissue Samples

For the study, we used 54 carotid artery specimens collected from 54 human patients who underwent carotid endarterectomy surgery. 15 samples were used for immunohistochemistry analysis, and 39 carotid arteries were used for the *in vitro* experiments. Written informed consent was received from the participants according to the Declaration of Helsinki. A pathologist examined the samples and classified them according to AHA guidelines. Type I (healthy), IV (atheromatous), and VI (complicated) lesions were selected for the study.

### 2.3. Mice

All the animal experiments were approved by the guidelines from Directive 2010/63/EU of the European Parliament on the protection of animals used for scientific purposes. Animal experiments performed in this study were approved by the Scientific and Research Ethics Committee of the Scientific Council of Health of the Hungarian Government under the registration number DE MÁB/157-5/2010 and are reported in accordance with the ARRIVE guidelines. C57BL/6 ApoE^−/−^ mice were maintained at the University of Debrecen under specific pathogen-free conditions in accordance with guidelines from Institutional Ethical Committee. To induce atherosclerotic plaque formation, standard chow diet was changed to atherogenic diet (15% fat, 1.25% cholesterol, ssniff-Spezialdiäten GmbH, Soest, Germany) at the age of 8 weeks. Mice were randomly divided into three groups, and parallel with the atherogenic diet, mice were injected intraperitoneally with NaSH (56 *μ*mol/kg body weight; *N* = 9), PPG (50 mg/kg, *N* = 5), or vehicle (PBS; *N* = 21) every other day as previously described [17]. Aortas were harvested after 8 weeks of treatment. All mice were euthanized by predictable and controllable administering slow-fill compressed CO_2_ asphyxiation.

### 2.4. Cell Culture

Human aortic endothelial cells (HAoECs) (PromoCell, Heidelberg, Germany) were cultured in medium 199 containing 15% FBS, antibiotics, L-glutamine, sodium pyruvate, and EC growth factor as described previously. HAoECs were used at passages 2 and 3 within 2 days postconfluence. Human aortic smooth muscle cells (HASMCs) (PromoCell, Heidelberg, Germany) were cultured in DMEM supplemented with 10% FBS, L-glutamine, sodium pyruvate, and antibiotics. RAW264.7 murine macrophages (ATCC) were grown in RPMI supplemented with L-glutamine, sodium pyruvate, and antibiotics.

### 2.5. Immunohistochemistry

Immunohistochemistry from the carotid arteries was performed on formalin-fixed, paraffin-embedded tissue sections. 4 *μ*m slides were then deparaffinated using xylol and ethanol.

Samples were incubated with the anti-CSE primary monoclonal antibody (12217-1-AP Proteintech, Chicago, IL, USA) at a dilution of 1 : 200. Other slides of the same samples were incubated with antihemoglobin (clone: goat polyclonal HRP (ab19362), Proteintech Group, Rosemont, IL 60018, USA) primary monoclonal antibody at a dilution of 1 : 100; with HO-1 (clone: rabbit polyclonal (10701-1-AP), Proteintech Group, Rosemont, IL 60018, USA) primary monoclonal antibody at a dilution of 1 : 200; and with HO-2 (clone: rabbit polyclonal (14817-1-AP), Proteintech Group, Rosemont, IL 60018, USA) primary monoclonal antibody at a dilution of 1 : 400. Specific antibody binding was visualized by the Dako EnVision FLEX/HRP and FLEX DAB3 Chromogen detection system (Dako, Glostrup, Danmark) followed by hematoxylin counterstaining and coverage. The intensity and distribution of protein immunostaining were assessed by light microscopy (Leica DM2500 microscope, DFC 420 camera and Leica Application Suite V3 software, Leica).

### 2.6. Dual Immunohistochemistry

Dual IHC was done as follows. The CSE enzyme was specifically labeled with a polyclonal antibody clone (12217-1-AP, Proteintech, Manchester, UK; dilution 1 : 100) and detected by the EnVision Flex HRP/DAB+ (brown color) procedure. This was followed by a second incubation procedure with either of the primary antibodies (CD4, CD34, or SMA) which were detected by the Flex HRP system using the violet VIP chromogen (Vector Laboratories, Burlingame, CA) as a substrate. This setting clearly differentiated the DAB-related brown staining and the VIP-related violet staining even within the same cells by light microscopy. Methyl-green solution (Vector Laboratories, Burlingame, CA) was used to counterstain unlabeled cell nuclei of any of the tissue constituents.

### 2.7. Hemoglobin Preparation

Hb of different redox states, that is, (Fe^2+^) oxyHb, (Fe^3+^) metHb, and ferrylHb, were prepared as described [15]. Briefly, Hb was isolated from fresh blood drawn from healthy volunteers using ion-exchange chromatography on a DEAE Sepharose CL-6B column. MetHb was generated by incubation (30 min, 25°C) of purified Hb with a 1.5-fold molar excess of K_3_Fe(CN)_6_ over heme. FerrylHb was obtained by incubation (1 h, 37°C) of Hb with a 10 : 1 ratio of H_2_O_2_ to heme. After oxidation, both metHb and ferrylHb were dialyzed against saline (3 times for 3 hours at 4°C) and concentrated using Amicon Ultra centrifugal filter tubes (10,000 MWCO, Millipore Corp., Billerica, MA, USA). Aliquots were snap-frozen in liquid nitrogen, and stored at −80°C until use. The purity of each Hb preparation was evaluated by SDS-PAGE followed by silver staining. The purity of Hb preparations was above 99.9%. Hb concentrations were calculated as described by Winterbourn [21].

### 2.8. Isolation and Oxidation of LDL

LDL was isolated from the plasma of EDTA-anticoagulated venous blood of healthy volunteers by gradient ultracentrifugation (Beckman Coulter Inc., Brea, CA, USA). The density of plasma was adjusted to 1.3 g/mL with KBr and a two-layer gradient was made in a Quick-Seal ultracentrifuge tube by layering saline on 10 mL plasma. Ultracentrifugation was performed at 302,000*g* for 2 hours at 4°C (VTi 50.2 rotor). LDL samples were kept at −70°C until use, and the protein concentration was determined by Pierce BCA protein assay kit (Pierce Biotechnology, Rockford, IL, USA). LDL oxidation was carried out at 37°C in a reaction mixture containing LDL (200 *μ*g/mL), heme (5 *μ*mol/L), and H_2_O_2_ (75 *μ*mol/L).

### 2.9. Oxidation of LDL

LDL (200 *μ*g/mL) was oxidized with heme (5 *μ*mol/L) and H_2_O_2_ (75 *μ*mol/L) in the presence or absence of the sulfide donors NaSH, GYY4137, AP67, and AP72 at concentrations of 20 and 200 *μ*mol/L at 37°C. Conjugated diene formation was monitored continuously for 1 hour at 234 nm. Delta OD234 nm was calculated by subtracting optical density measured at the 0 time point from optical density measured at 1 hour. The formations of lipid hydroperoxides (LOOH) and thiobarbituric acid reactive substances (TBARS) were measured at 60 minutes following the initiation of lipid peroxidation. The method of Wolf was used to evaluate LOOH content in the LDL samples [14]. For the TBARS measurement, 50 *μ*L of a 200 *μ*g protein/mL LDL sample was mixed with 100 *μ*L of thiobarbituric acid reagent (0.375 g 2-thiobarbituric acid, 2.08 mL HCl, and 15 mL 10% trichloroacetic acid to a final volume of 100 mL). After heating at 90°C for 20 minutes, the samples were cooled and extracted with 200 *μ*L n-butanol. The upper phase was measured spectrophotometrically at 532 nm. Results were calculated using a molar extinction coefficient of 1.56 × 10^5^ M^−1^·cm^−1^ and are expressed as nmol TBARs/mg protein.

### 2.10. Plaque Lipid Oxidation

Lipids were extracted from human carotid artery plaques as described previously [13]. Plaque lipids (0.5 mg/mL) were incubated with Hb (100 *μ*mol/L) in the presence or absence of the sulfide donors NaSH, GYY4137, AP67, and AP72 at 200 *μ*mol/L concentration for 4 days at 37°C. In other cases, complicated lesions containing intraplaque hemorrhage were homogenized in saline. These samples (0.5 mg/mL) were incubated at 37°C for 3 days in the presence or absence of the sulfide donors NaSH, GYY4137, AP67, and AP72. Lipid peroxidation was assessed by measuring LOOH and TBARs.

### 2.11. Hb Oxidation and Detection of Covalently Cross-Linked Hb Species

Purified Hb (5 *μ*mol/L heme) was incubated with H_2_O_2_ (25 *μ*mol/L) or oxidized LDL (50 *μ*g protein/mL) in the presence or absence of the sulfide donors NaSH, GYY4137, AP67, and AP72 at 37°C for 1 hour. For the detection of the covalently cross-linked Hb species, 0.5 *μ*g of Hb samples were applied to 12.5% SDS-PAGE gels. After electrophoresis, proteins were transferred to a nitrocellulose membrane (Amersham Biosciences Corp., Piscataway, NJ, USA) and Hb was identified using an HRP-conjugated goat anti-human Hb polyclonal antibody (ab19362-1 Abcam, Cambridge, UK) at a dilution of 1 : 15,000.

### 2.12. Stopped-Flow Spectrophotometry

Kinetic measurements were performed with a sequential stopped-flow apparatus (DX-18 MV, Applied Photophysics Ltd., Leatherhead, UK) using a 150 W Xe arc lamp. The reactions were followed at *λ* = 406, 425, 570, and 620 nm. All kinetic traces were collected in 20 mmol/L phosphate buffer at pH 7.40 at 25°C. At least 3 kinetic runs were made and averaged at each concentration to establish the corresponding kinetic traces.

### 2.13. Endothelial Cell Cytotoxicity Assay

LDL (200 *μ*g/mL) was oxidized with heme (5 *μ*mol/L) and H_2_O_2_ (75 *μ*mol/L). Oxidized LDL was incubated at 37°C overnight with the sulfide donors NaSH, GYY4137, AP67, and AP72 at concentrations of 20 and 200 *μ*mol/L. Confluent HAoECs grown in 96-well tissue culture plates were washed twice with PBS and exposed to oxLDL samples for 6 hours. Cell viability was assessed by MTT assay as described previously [22].

### 2.14. Endothelial Cell Monolayer Integrity Assay

Electric cell substrate impedance sensing method was used to measure endothelial monolayer integrity. HAoECs were cultured on 8-well electrode arrays (8W10E, Applied BioPhysics Inc., Troy, NY, USA). Upon confluence, cells were challenged with Hb (10 *μ*mol/L) oxidized with H_2_O_2_ (50 *μ*mol/L) in the presence of sulfide donor molecules at the concentration of 200 *μ*mol/L. The complex impedance spectrum was monitored with an ECIS Zᴓinstrument (Applied BioPhysics Inc., Troy, NY, USA) for 3 hours in every minute. Intercellular gap formation was calculated based on the difference between monolayer resistance at 4000 Hz at the 0 time point and 3 hours. In other experiments, HAoECs were treated with Hb (20 *μ*mol/L) oxidized with plaque lipids (400 *μ*g/mL) in the presence of sulfide donor molecules (200 *μ*mol/L) and impedance spectrum was monitored for 12 hours. Intercellular gap formation was calculated based on the difference between monolayer resistance at 4000 Hz at the 0 time point and 12 hours.

### 2.15. Western Blot

The cells were cultured in 6-well plates, and upon reaching the confluence, the cells were treated with different triggers. After 8 hours of treatment, the cells were solubilized in protein lysis buffer containing 10 mmol/L Tris-HCl, 5 mmol/L EDTA, 150 mmol/L NaCl (pH 7.2), 1% Triton X-100, 0.5% Nonidet P-40, and protease inhibitors (Complete Mini, F. Hoffmann-La Roche Ltd., Basel, Switzerland). In other experiments, tissue samples were homogenized under liquid nitrogen and solubilized in protein lysis buffer. Proteins (10–20 *μ*g) were applied to 12.5% SDS-PAGE gels. After electrophoresis, proteins were transferred to a nitrocellulose membrane (Amersham Biosciences Corp., Piscataway, NJ, USA). Proteins were identified using the following antibodies: mouse anti-human HO-1 antibody (Calbiochem, San Diego, CA, USA, 374087, dilution 1 : 2500), rabbit anti-human HO-2 antibody (Proteintech, Chicago, IL, USA, 14817-1-AP), rabbit anti-human CSE antibody (Proteintech, Chicago, IL, USA, 12217-1-AP, dilution 1 : 1000), rabbit anti-human VCAM-1 (Santa Cruz Biotechnology Inc., Dallas, TX, USA, sc8304, dilution 1 : 200), mouse anti-human GAPDH (Novus Biologicals, Littleton, CO, USA NB-300-221, dilution 1 : 1000), anti-rabbit IgG HRP-conjugate (GE Healthcare Life Sciences, Piscataway, NJ, USA, NA934, dilution 1 : 15,000), and anti-mouse IgG HRP-conjugate (GE Healthcare Life Sciences, Piscataway, NJ, USA, NA931, dilution 1 : 15,000). Antigen-antibody complex was detected by a horseradish peroxidase chemiluminescence system according to the manufacturer's instructions (GE Healthcare Life Sciences, Piscataway, NJ, USA). Quantification was performed using video densitometry (AlphaDigiDoc RT, Alpha Innotech Corp., San Leandro, CA, USA).

### 2.16. Quantitative Real-Time PCR (qRT-PCR)

ApoE^−/−^ mice were intraperitoneally injected with NaSH (56 *μ*mol/kg body weight) or vehicle (PBS) in every other day over 8 weeks. Parallel with the treatment, the mice were fed with atherogenic diet. Control mice were fed with standard chow diet. After, the mice were sacrificed and aortas were harvested. Total RNA was isolated using RNAzol STAT-60 according to the manufacturer's instructions (cat. number Tl-4120, TEL-TEST Inc., Friendswood, TX, USA). RNA concentration was measured with NanoDropTM 2000c spectrophotometer (cat. number S06497c, Thermo Scientific Inc., Waltham, MA, USA). After that, cDNA synthesis was performed using a high-capacity cDNA kit (cat. number 43-688-13, Applied Biosystems, Foster City, CA). We used real-time PCR technique for quantification of mRNA levels of HO-1 (Mm00516005_m1, Thermo Fisher Scientific Inc.) and beta-actin (Mm02619580_g1, Thermo Fisher Scientific Inc.). TaqMan Universal PCR Master Mix was purchased from Applied Biosystems (cat. number 4269510, Applied Biosystems, Foster City, CA). Finally, we performed TaqMan quantitative PCR (40 cycles at 95°C for 15 sec. and 60°C for 1 min.) in 96-well plates with the Bio-Rad CFX96 (Bio-Rad Laboratories Inc., Hercules, California, USA) detection system. Results were expressed as mRNA expression normalized to beta-actin.

### 2.17. Determination of Sulfide Level from Tissue with Zinc Precipitation Assay

Sulfide levels were measured with zinc precipitation method based on a method developed by Gilboa-Garber [23] and modified by Ang et al. [24]. The human carotid artery was homogenized under liquid nitrogen in 7.4 pH PBS and was sonicated. After that, the sample was centrifuged at 12,000*g* for 15 min and the lipid-free clear supernatant was collected. 200 *μ*L sample was mixed with 350 *μ*L 1% zinc acetate and 50 *μ*L 1.5 mol/L sodium hydroxide and incubated for 60 minutes on a shaker. Incubation step was followed by centrifugation at 2000*g* for 5 minutes to pellet the generated zinc sulfide. The supernatant was then removed, and the pellet washed with 1 mL of distilled water by vortexing extensively, followed by centrifugation at 2000*g* for 5 minutes. The supernatant was then aspirated off and the pellet reconstituted with 160 *μ*L of distilled water and mixed with 40 *μ*L of premixed dye (20 *μ*L of 20 mmol/L dimethyl-p-phenylenediamine dihydrochloride (NNDP) in 7.2 mol/L hydrochloric acid (HCl) and 20 *μ*L of 30 mmol/L iron(III) chloride (FeCl_3_) in 1.2 mol/L HCl). After 10 min, the absorbance of the generated methylene blue (MB) was measured with spectrophotometer at 667 nm. Since during the reaction 1 mol/L MB formed from 1 mol/L sulfide, the concentration was determined by the MB's extinction coefficient (30,200 M^−1^·cm^−1^). Samples were normalized for protein concentration.

### 2.18. Experimental Units

“*N*” represents the number of tissue samples used in each group. “*n*” denotes the number of replications of the independent results.

### 2.19. Study Approval

Collection of carotid artery plaques from patients who underwent carotid endarterectomy surgery was approved by the Scientific and Research Ethics Committee of the Scientific Council of Health of the Hungarian Government under the registration number of DE OEC RKEB/IKEB 3712-2012.

### 2.20. Statistics

Data were analyzed by GraphPad Prism 5.02 software (GraphPad Software Inc., 7825 Fay Avenue, Suite 230 La Jolla, CA 92037). All statistics data are expressed as mean ± sem. Differences in means were analyzed by Student's *t*-test or one-way ANOVA with Dunnett's post test as appropriate. *p* < 0.05 was considered significant.

## 3. Results

### 3.1. CSE Is Upregulated in Human and Mouse Atheromatous Plaques

**Figure 1:**
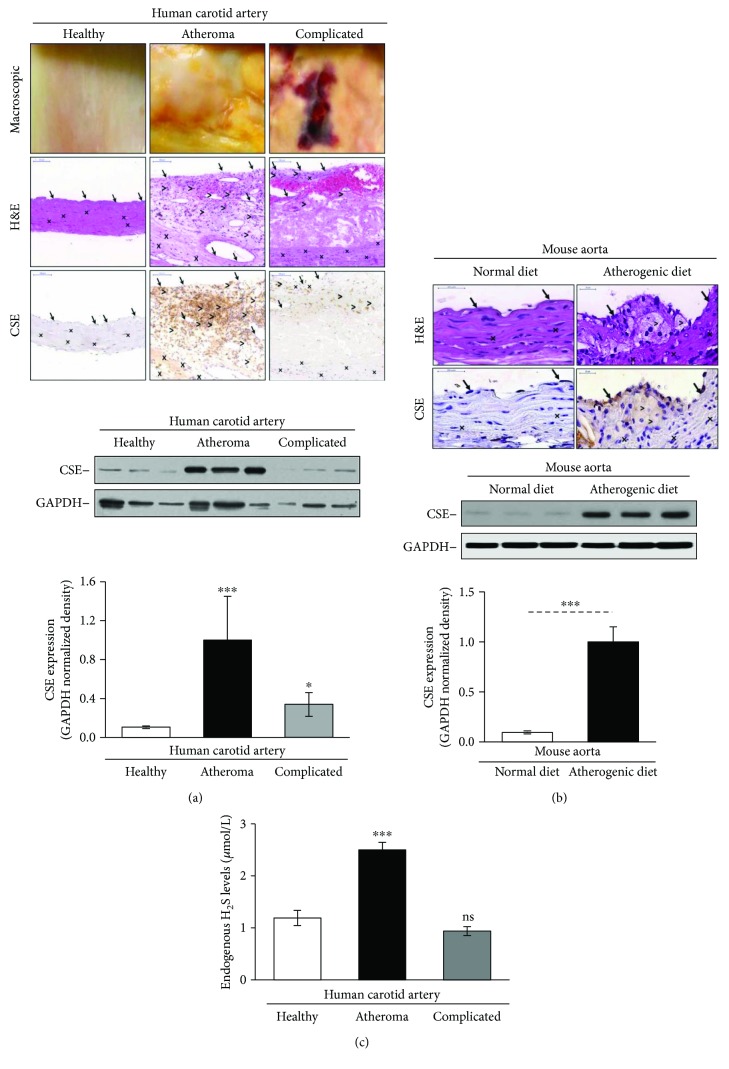
CSE is markedly upregulated in atheromatous lesions. (a, upper panels) Immunohistochemistry detection of the CSE expression in human carotid artery specimens. Macroscopic pictures, H&E, and CSE staining are shown. Magnification of the human histology samples was 150x. Arrows indicate endothelial cells, crosses show smooth muscle cells, and comparison signs mark macrophages. (a, lower panels) Pieces of carotid artery representing the healthy vessel; atheromatous and complicated lesions were selected by macroscopic examination. Representative Western blot showing CSE expression in carotid artery tissue lysates (20 *μ*g/lane) (*N* = 3, three human carotid artery specimens from three patients in each group). Immunoblots were reprobed with GAPDH. The bar graph shows CSE expression normalized to GAPDH. ^∗∗∗^ *p* < 0.005 and ^∗^ *p* < 0.05 compared to healthy samples. (b) ApoE^−/−^ mice were kept on normal chow diet or atherogenic diet for 8 weeks. (b, upper panels) H&E and CSE staining are shown. Magnification of the mice aortas was 700x. Arrows indicate endothelial cells, crosses show smooth muscle cells, and comparison signs mark macrophages. (b, lower panels) Representative Western blot showing CSE expressions of carotid artery samples (10 *μ*g/lane) (*n* = 3, each performed in triplicate). Immunoblots were reprobed with GAPDH. The bar graph shows CSE expression normalized to GAPDH. (c) Endogenous H_2_S levels in different types of the carotid arteries (*N* = 6).

**Figure 2:**
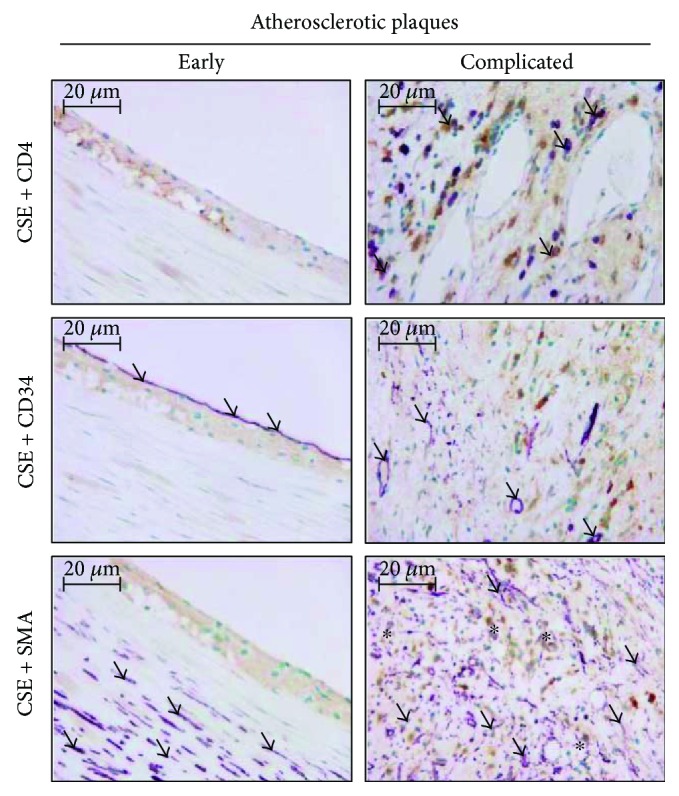
Double immunohistochemistry is demonstrating CSE expression in different cellular compartments of early (left) and complicated (right) atherosclerotic plaques of the carotid artery. Upper panels: CSE (DAB, brown color) and CD4 (VIP, violet) highlighting tissue macrophages (arrows); middle panels: CSE (DAB, brown color) and CD34 (VIP, violet) presenting endothelial cells (arrows); and lower panels: CSE (DAB, brown color) and smooth-muscle actin (VIP, violet) referring to smooth-muscle cells (arrows) and myofibroblasts (asterisk) (×400 magnification).

**Figure 3:**
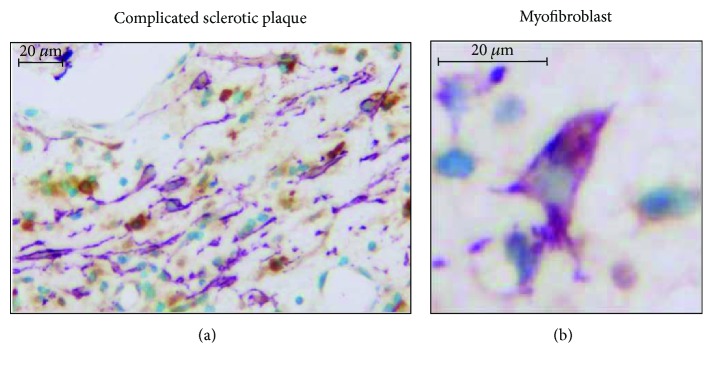
CSE expression in activated myofibroblasts in a cellular area of a complicated sclerotic plaque. (a) SMA+ myofibroblasts (VIP, violet) partially coexpress CSE (DAB, brown staining) in their cytoplasm. Additional CSE labeling can be observed in tissue macrophages (brown only) (×400 magnification). (b) A single enlarged SMA+ fibroblast-type cell with cytoplasmic brown CSE-related colabeling (×1000 magnification).

To investigate whether CSE expression changes during atherogenesis, we performed both immunostainings of human carotid artery specimens and Western blotting. Western blot analysis of human vessel samples revealed that atheromatous lesions exhibit the higher content of CSE as compared to the healthy artery, or complicated lesions (Figure 1(a), lower panels). Localization of CSE by immunostaining (Figure 1(a), upper panels) demonstrated that CSE was highly expressed in macrophages, and foam cells of atheromatous plaque as well as in macrophages and foam cells of complicated lesions showing visible evidence of intraplaque hemorrhage (Figure 1(a), upper panel). For better visibility, we present same histological pictures with higher magnification as Supplementary Figure 1. We found that CSE-positive cells were more common in the atheroma compared to the complicated lesions. To evaluate CSE expression more accurately in different cell types of human samples, we performed double staining with cellular markers for macrophages, endothelial cells, and smooth muscle cells (Figure 2). Double immunohistochemistry revealed tissue macrophages (violet) presenting CSE expression (brown color) in complicated lesions (upper panels). Endothelium (middle panels, violet) was also stained for CSE (middle panels, brown color). Importantly, we identified myofibroblasts in complicated lesions expressing high CSE level (Figure 3). SMA-positive myofibroblasts (violet) coexpressing CSE (brown color) in their cytoplasm were abundant.

To further support this observation, we compared CSE expression in the aorta of ApoE^−/−^ mice fed with normal or atherogenic diet for 8 weeks. Mice fed on the atherogenic diet provoked extensive atherosclerotic plaque formation which was associated with elevated expression of CSE as demonstrated by Western blot (Figure 1(b), lower panels). CSE was localized in endothelium, macrophages, and foam cells as revealed by immunohistochemistry (Figure 1(b), upper panel). For improved visibility, we show the same histological pictures with higher magnification as Supplementary Figure 2.

Furthermore, we investigated the endogenous sulfide levels in different types of the carotid arteries and found increased sulfide levels in atheroma compared to healthy vessel and complicated lesion (Figure 1(c)).

### 3.2. Sulfide Inhibited Lipid Peroxidation *In Vitro* and *In Vivo*

**Figure 4:**
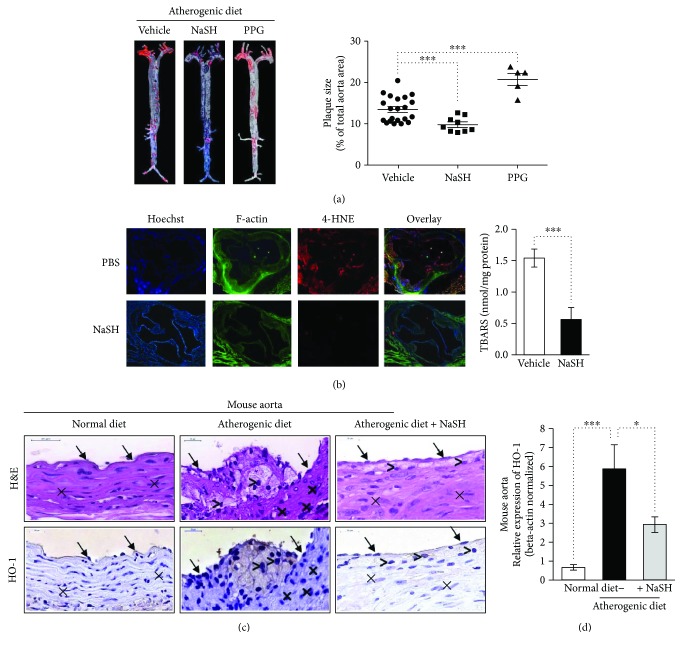
Sulfide inhibits lipid-peroxidation *in vivo* and *in vitro*. (a, left panel) Representative oil red O staining of aorta derived from ApoE^−/−^ mice fed with atherogenic diet and treated with NaSH (*N* = 9), PPG (*N* = 5), or vehicle (PBS, *N* = 21) for 8 weeks. (a, right panel) Quantification of atherosclerotic plaque size was performed with ImageJ software. (b, left panel) Representative aortic sections (6 *μ*m) stained for F-actin (phalloidin-FITC, green), 4-HNE (red), and DNA (Hoechst, blue) (*N* = 9). (b, right panel) TBARS content of aorta derived from ApoE^−/−^ mice fed with atherogenic diet and treated with vehicle (*N* = 9) or NaSH for 8 weeks (*N* = 9). (c) ApoE^−/−^ mice were fed with regular diet (*N* = 9) or atherogenic diet for 8 weeks. Parallel with the atherogenic diet, the mice were kept intraperitoneally vehicle (*N* = 9) or NaSH (*N* = 9). Immunohistology was showing hematoxylin and eosin and HO-1 staining of mice aorta samples. Endothelial cells are indicated by arrows, smooth muscle cells were marked by cross, and macrophages were marked by comparison signs. The magnification of the mice aortas was 700x. (d) Real-time qPCR was showing HO-1 relative expression in mouse aorta. ∗ indicates statistical significance (*p* < 0.05). ∗∗∗ indicates statistical significance (*p* < 0.0001).

In accordance with previous findings [17], we observed that administration of NaSH solution reduced, whereas inhibition of CSE activity by DL-propargylglycine (PPG) increased atherogenic diet-induced atherosclerotic plaque formation in ApoE^−/−^ mice (Figure 4(a), left and right panels). Therefore, we examined the extent of oxidative injury by performing immunofluorescence staining for the cytotoxic lipid peroxidation product 4-hydroxynonenal (4-HNE) in aortic root in a vehicle or NaSH-treated ApoE^−/−^ mice. Strong positive 4-HNE immunostaining was observed in aorta from vehicle-treated mice, whereas 4-HNE level was markedly lower in aorta from NaSH-treated mice (Figure 4(b), left panel). To further confirm that lipid peroxidation was inhibited by sulfide, we measured thiobarbituric acid reactive substance (TBARS) content of the aorta derived from a vehicle or NaSH-treated ApoE^−/−^ mice. TBARS content of sulfide-treated aorta was approximately one-third of the vehicle-treated control samples (Figure 4(b), right panel). Since heme oxygenase-1 (CSE) was shown to be induced by lipid hydroperoxide [25], we also tested whether sulfide alters HO-1 expression in an atherosclerotic mouse model. HO-1 was strongly upregulated in the aorta of ApoE^−/−^ mice fed with atherogenic diet and it was mainly expressed by endothelial cell and macrophages (Figure 4(c)). We found that HO-1 mRNA level was reduced in NaSH-treated mice, presumably due to the attenuated oxidative stress (Figure 4(d)). Next, we examined whether sulfide releasing molecules could prevent lipid peroxidation of lipids derived from human atheromatous lesions *in vitro*. We provoked lipid peroxidation *in vitro* with heme or hemoglobin (Hb) to model intraplaque hemorrhage. Heme induced a robust increase in lipid peroxides (LOOH) and TBARS content of plaque lipids which was inhibited by all sulfide donors (Suppl. Figures 3A and 3B). Similarly, sulfide-releasing molecules attenuated Hb-mediated formation of both LOOH and TBARS (Suppl. Figures 3C and 3D). We also checked whether polysulfides and decomposed sulfide donors alter lipid peroxidation *in vitro*. Neither polysulfide nor decomposed sulfide donors were able to block lipid peroxidation (Suppl. Figure 5).

### 3.3. Sulfide Inhibited the Formation of Lipid-Peroxidation Products in Human Hemorrhaged Lesions

Next, we examined whether the different H_2_S-releasing compounds could prevent hemorrhage-mediated lipid-peroxidation upon intraplaque hemorrhage. Human carotid endarterectomy specimens with obvious macroscopic evidence of intraplaque hemorrhage (Suppl. Figure 4A) were homogenized and incubated at 37°C in the presence or absence of the sulfide donors (200 *μ*mol/L). We determined LOOH and TBARS content of the samples at day 0 and day 4. During the four-day incubation, LOOH content increased about 2.7-fold, whereas the level of TBARS elevated by 2.4-fold (Suppl. Figures 4B and 4C). The formation of both LOOH and TBARS were attenuated by the sulfide donors (Suppl. Figures 4B and 4C). Moreover, AP67 and AP72 were able to decrease LOOH content below the basal LOOH level measured at day 0 (Suppl. Figure 4B).

### 3.4. Sulfide Donor Molecules Inhibit Oxidative Cross-Linking of Hb Subunits

**Figure 5:**
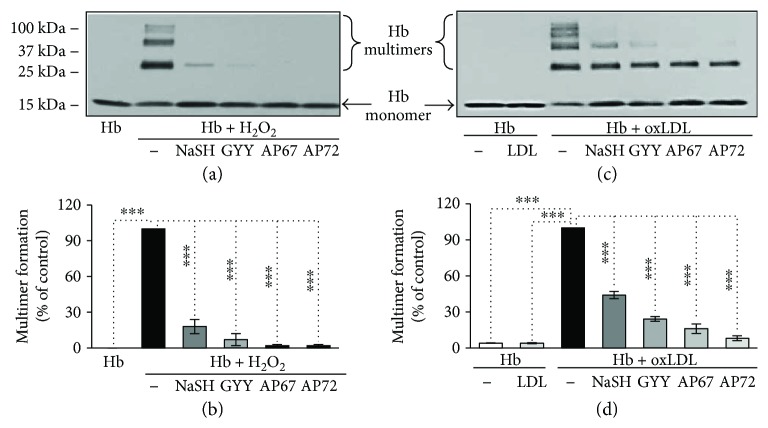
Sulfide inhibits the formation of covalently cross-linked Hb multimers. (a) Hb (5 *μ*mol/L) was incubated with H_2_O_2_ (75 *μ*mol/L) in the presence or absence of the H_2_S donors NaSH, GYY4137, AP67, and AP72 (200 *μ*mol/L) at 37°C for 90 minutes. Samples (0.5 *μ*g Hb) were subjected to SDS-PAGE and Hb species were detected by Western blotting. Representative experiment, *n* = 3. (b and d) Quantification of the multimer formation was showed. (c) Hb (5 *μ*mol/L) was incubated with oxLDL (50 *μ*g/ml) in the presence or absence of the sulfide donors NaSH, GYY4137, AP67, and AP72 (200 *μ*mol/L) at 37°C for 4 hours. Samples (0.5 *μ*g Hb) were subjected to SDS-PAGE and Hb species were detected by Western blotting. Representative experiment, *n* = 3. ∗∗∗ indicates statistical significance (*p* < 0.0001).

Covalent cross-linking of Hb occurs in advanced atherosclerotic lesions. To model the oxidative environment present in the atherosclerotic plaque *in vitro*, we used H_2_O_2_ (25 *μ*mol/L) or oxLDL (50 *μ*g/mL) to trigger such oxidative cross-linking of Hb (5 *μ*mol/L) in the presence or absence of sulfide donors. Covalently cross-linked Hb species were subsequently detected by Western blot analysis. The formations of dimeric, tetrameric, and multimeric Hb species were detected in both H_2_O_2_ and oxLDL-treated Hb samples. At a concentration of 200 *μ*mol/L, all the sulfide-generating molecules significantly inhibited the formation of covalently cross-linked Hb multimers triggered by either H_2_O_2_ or oxLDL (Figures 5(a)–5(d)). AP67 and AP72 provided complete inhibition against H_2_O_2_-induced Hb oxidation, and these donors were also the most effective at inhibiting oxLDL-mediated Hb oxidation (Figures 5(a)–5(d)).

### 3.5. Reactions of FerrylHb with Sulfide

**Figure 6:**
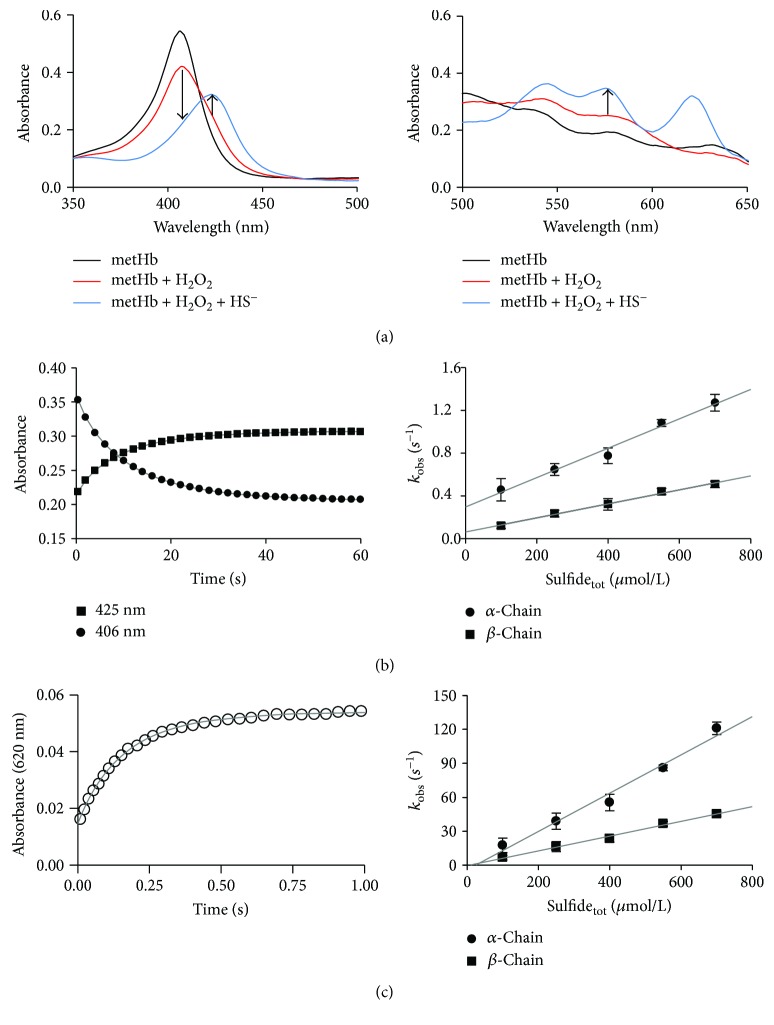
Reactions of ferrylHb with sulfide. (a, left and right panels) Representative UV-vis spectra of 4 *μ*mol/L metHb (black line), reaction of 4 *μ*mol/L metHb with 8 *μ*mol/L H_2_O_2_ after 400 s (red line) and reaction of 4 *μ*mol/L ferrylHb with 100 *μ*mol/L sulfide after an additional 60 s (blue line). Arrows indicate wavelength values where the kinetic runs were recorded. (b, left panel) Representative stopped-flow kinetics traces for the reaction of 4 *μ*mol/L ferrylHb with 100 *μ*mol/L sulfide at 406 nm (circles) and 425 nm (squares) and the corresponding double exponential fits (solid lines). (b, right panel) Sulfide concentration dependencies of the obtained pseudo-first-order rate constants from the double exponential fits of the stopped-flow kinetic traces (*λ* = 425 nm). MetHb (4 *μ*mol/L) was reacted with H_2_O_2_ (8 or 20 *μ*mol/L) in the presence of sulfide (100–700 *μ*mol/L). The faster (circles) and slower (squares) reactions represent reactions of the ferryl moieties of the alpha and beta chains, respectively. Data points and error bars represent the average and standard deviations of 4 independent experiments. (c, left panel) Representative stopped-flow kinetics traces for the reaction of 4 *μ*mol/L ferrylHb with 100 *μ*mol/L sulfide at 620 nm (blank circles) and the corresponding double exponential fit (solid lines). (c, right panel) Sulfide concentration dependence of the pseudo-first-order rate constant for the reaction of ferrylHb with sulfide from the double exponential fits of the kinetic traces, where ferrylHb was generated in the reaction of 8 *μ*mol/L metHb with 40 *μ*mol/L H_2_O_2_ in the first mixing cycle of a sequential stopped-flow experiment, followed by mixing with sulfide solutions in a 1 : 1 ratio in the second mixing cycle after a 240 s delay time. The faster (circles) and slower (squares) reactions represent the reactions of the ferryl moieties of the alpha and the beta chains, respectively. Data points and error bars represent the average and standard deviations of 3 independent experiments.

To test the possibility that the observed protective effects of sulfide donors were due to the reduction of ferrylHb, we next investigated the kinetics of the reaction of ferrylHb species with sulfide in a cell-free system using stopped-flow spectrophotometry. Initially, ferrylHb was prepared in situ by the reaction of metHb with 5 molar equivalents of H_2_O_2_ in the first mixing cycle of a sequential stopped-flow experiment. Under our experimental conditions, the formation of ferrylHb was complete in 400 s and resulted in spectral transitions (see the 350–500 and 500–650 nm ranges on Figure 5(a), left and right panels) that were characteristic to the formation of ferrylHb. Therefore, sulfide was reacted with ferrylHb in the second mixing cycle using a delay time of 400 s. Rapid changes in the UV-vis spectra predicted a favorable reaction between sulfide and ferrylHb under these conditions. The appearance of a new peak at 620 nm together with the shift of the Soret band at 400 nm and the absorbance increases at 530 and 580 nm were indicative of the formation of sulfhemoglobin (Figure 6(a), left and right panels). Kinetic traces were measured at a >10-fold excess of sulfide over ferrylHb to maintain pseudo-first-order conditions and recorded initially at 406, 425, and 570 nm (Figure 6(b), left and right panels). Under these conditions, they exhibited a biexponential character, which most likely corresponded to separated reactions of the alpha and beta chain ferryl heme centers (faster and slower phase, resp.) as shown before. The obtained pseudo-first-order rate constants from the double exponential fits of the kinetic traces at 406, 425, and 570 nm show similar linear correlations with the applied sulfide concentrations, indicating first-order dependencies of the rate law on both ferrylHb and sulfide concentrations (Figure 6(b), right panel). Therefore, the apparent second-order rate constants at pH 7.4 were calculated from the slopes of the lines on Figure 6(b), right panel, to be (1.43 ± 0.06) × 10^3^ M^−1^·s^−1^ and (6.5 ± 0.2) × 10^2^ M^−1^·s^−1^ for the alpha and beta chains, respectively. The previous report proposed that related ferrylHb species are generated in the reactions of oxyHb with H_2_O_2_ as with metHb [26]. Indeed, we observed similar kinetics with sulfide for the ferrylHb species that were produced in the reaction of oxyHb with H_2_O_2_ as the ones described above for metHb (data not shown).

However, kinetic traces that were recorded at 620 nm (representing the formation of sulfheme Hb) (Figure 6(c), left panel) indicated two orders of magnitude faster rates (also exhibiting double-exponential behaviors with second-order rate constants of (1.7 ± 0.1) × 10^5^ and (6.5 ± 0.3) × 10^4^ M^−1^·s^−1^) (Figure 6(c), right panel). We obtained preliminary kinetic evidence that the relatively slow reactions recorded at 425 nm and the 2 orders of magnitude faster reactions at 620 nm were parallel and not consecutive reactions. As mentioned above, ferrylHb represents a mixture of oxidized Hb species, where an unpaired electron is located at different sites of the protein [27]. Therefore, we propose that the observed kinetically isolated reactions were due to different ferrylHb species reacting with sulfide. We are currently investigating the chemical nature of these ferrylHb species, but their characterization is outside the scope of the present study.

### 3.6. Hb-Lipid Interactions and Subsequent Endothelial Responses Are Attenuated by Sulfide

**Figure 7:**
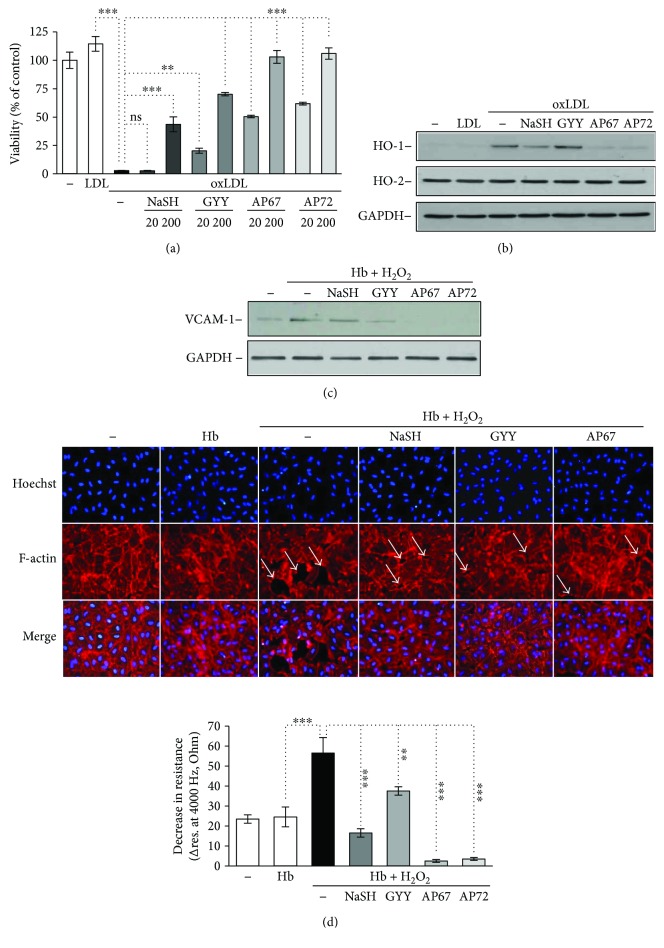
Endothelial responses provoked by Hb-lipid interactions are attenuated by sulfide donors. (a) OxLDL (200 *μ*g/mL) was incubated with sulfide donors NaSH, GYY4137, AP67, and AP72 at the concentrations of 20 and 200 *μ*mol/L at 37°C for 24 hours. Confluence HAoECs were exposed to the LDL samples for 4 hours then cell viability was determined by MTT assay. Representative experiment, *n* = 3, each performed in 8 wells in parallel. (b) OxLDL (200 *μ*g/mL) was incubated with sulfide donors NaSH, GYY4137, AP67, and AP72 at the concentration of 20 and 200 *μ*mol/L at 37°C for 24 hours. Confluence HAoECs were exposed to LDL samples (50 *μ*g/mL) for 8 hours. HO-1 and HO-2 protein expressions were determined by Western blotting. Representative experiment, *n* = 3. (c–e) Hb (10 *μ*mol/L) was incubated with H_2_O_2_ (50 *μ*mol/L) in the presence or absence of sulfide donors NaSH, GYY4137, AP67, and AP72 (200 *μ*mol/L) at 37°C for 90 minutes. (c) Confluence HAoECs were exposed to the obtained Hb samples for 8 hours and VCAM-1 expression was determined by Western blotting. Representative experiment, *n* = 3. (d, lower panel) Confluence HAoECs cultured in ECIS plates were exposed to the obtained Hb samples and transendothelial electrical resistance was monitored by ECIS instrument for 3 hours. Representative experiment, *n* = 3, each performed in triplicate (d, upper panel). HAoECs grown on coverslips, upon reaching confluence cells were exposed to the Hb samples for 8 hours. Cells were stained for F-actin (phalloidin-TRITC, red) and DNA (Hoechst, blue). White arrows show intercellular gaps. Representative image, *n* = 5. ∗∗ indicates statistical significance (*p* < 0.001). ∗∗∗ indicates statistical significance (*p* < 0.0001).

Interactions between Hb and lipids upon intraplaque hemorrhage result in the formation of reactive lipid mediators as well as oxidized Hb species. These molecules trigger oxidative stress and cell activation, respectively. To investigate whether sulfide could attenuate these harmful reactions, we treated oxidized LDL with sulfide donors for 4 hours. Then, HAoECs were exposed to the LDL samples for 6 hours. Oxidized LDL decreased cell viability by about 97% (Figure 7(a)). Pretreatment of oxLDL with sulfide donors at the concentration of 200 *μ*mol/L significantly attenuated oxLDL induced to HAoECs (Figure 7(a)). AP67 and AP72 were more potent at inhibiting LDL-induced cytotoxicity presumably because they generate H_2_S more efficiently than GYY4137 [19] (Figure 7(a)). Sublethal concentrations of oxLDL induced the expression of heme oxygenase-1 (HO-1), a stress-responsive protein in HAoECs (Figure 7(b)). We found that HO-1 expression provoked by a sublethal dose of oxLDL was suppressed by AP67 and AP72 but not by NaSH and GYY4137 in HAoECs (Figure 7(b)). We also tested the constitutive heme oxygenase-2 (HO-2) expression in these samples and we found that HO-2 level remained unchanged (Figure 7(b)). H_2_S donors at concentrations we used did not enhance the level of HO-1 and HO-2 in HAoECs (data not shown). Thus, in our system, heme oxygenases were not involved in the cellular responses provided by H_2_S. Oxidized Hb species in particular ferrylHb trigger endothelial cell activation, characterized by elevated expression of adhesion molecules and increased intercellular permeability. Therefore, we next examined whether sulfide donor molecules could modulate endothelial responses via inhibiting ferrylHb formation. Hb was treated with H_2_O_2_ in the absence or presence of the sulfide releasing molecules. Confluent HAoECs were then treated with the obtained samples, and VCAM-1 expression as a marker of endothelial cell activation, endothelial monolayer resistance, and intercellular gap formation was investigated.

We found that Hb samples treated with H_2_O_2_ in the presence of slow sulfide-releasing molecules were able to attenuate VCAM-1 expression, while NaSH could not diminish it (Figure 7(c)). Then, we investigated the integrity of HAoECs monolayers. Native Hb did not change HAoECs monolayer resistance compared to control cells during the 3-hour treatment (Figure 7(d), lower panel), but when HAoECs monolayers were exposed to oxidized Hb, monolayer resistance was significantly decreased (Figure 7(d), lower panel), suggesting that oxidized Hb provoked intercellular gap formation in accordance with previous observations [11, 15]. Importantly, each sulfide-releasing molecule attenuated the decline of monolayer resistance (Figure 7(d), lower panel) presumably via the inhibition of ferrylHb formation. Moreover, AP67 and AP72 not only inhibited the oxHb-mediated decrease in monolayer resistance but also increased resistance significantly above the nontreated HAoECs monolayer's resistance (Figure 7(d), lower panel). To confirm that the observed decrease in monolayer resistance upon treatment of HAoECs with oxidized Hb was due to intercellular gap formation, we performed immunostaining on cells after oxidized Hb exposure (Figure 7(d), upper panel). Accordingly, in the present studies, endothelium exposed to oxHb exhibited gaps between the cells visualized by F-actin staining (Figure 7(d), upper panel). As demonstrated in panel E, rearrangement of F-actin cytoskeleton and subsequently the formation of intercellular gaps were prevented by NaSH, GYY4137, and AP67. We also tested if polysulfides and decomposed sulfide donors could affect endothelial responses. Interestingly, we found that polysulfides, but not decomposed sulfide donors, were able to inhibit oxLDL-induced cell death (Suppl. Figures 6A and 6B). Furthermore, increased expression of HO-1 and VCAM-1 provoked by oxLDL or oxHb at a sublethal dose was not altered by polysulfides and decomposed sulfide donors (Suppl. Figures 6C and 6D).

### 3.7. Atherogenic Lipids and Proinflammatory Cytokines Increased the Expression of CSE in Diverse Cells of the Atherosclerotic Vessel Wall

**Figure 8:**
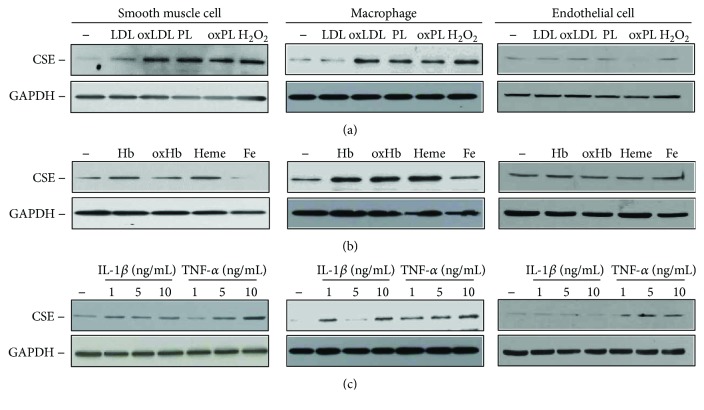
Modulators of CSE expression in HASMCs, macrophages, and endothelial cells. (a) Confluent HASMCs, RAW264.7 cells, and HAoECs were exposed to LDL (50 *μ*g/mL), oxLDL (50 *μ*g/mL), PL (500 *μ*g/mL), oxPL (500 *μ*g/mL), and H_2_O_2_ (100 *μ*mol/L) for 8 hours. (b) Confluent HASMCs, RAW264.7 cells, and HAoECs were exposed to Hb, oxHb, heme (50 *μ*mol/L heme group each), and Fe-ammonium citrate (50 *μ*mol/L) for 8 hours. (c) Confluent HASMCs, RAW264.7 cells, and HAoECs were exposed to IL-1*β* (1–5-10 ng/mL) or TNF-*α* (1–5-10 ng/mL) for 8 hours. Representative Western blots showing CSE expressions of cell lysates (20 *μ*g/lane) (*n* = 3). Immunoblots were reprobed with GAPDH.

To identify triggers and cell types responsible for altered CSE expression in the atherosclerotic vessel wall, we next exposed HASMCs, macrophages, and HAoECs to different agonists considered relevant in atherogenesis. First, we examined the effect of lipids, for example, LDL and plaque lipids extracted from human aorta specimens with lipid-rich atheromatous plaques. We challenged the cells with native and oxidized forms of these lipids and applied H_2_O_2_ as a positive control (Figure 8(a)). We found that all the three cell types expressed CSE and that H_2_O_2_ exposure upregulated the expression of this H_2_S synthesizing enzyme in HASMCs and macrophages. Both HASMCs and macrophages responded to oxLDL, native, and oxidized plaque lipids by upregulating the expression of CSE (Figure 8(a), left and middle panels). Next, we challenged the cells with different forms of Hb and Hb breakdown products such as heme and iron which are known to accumulate in the atherosclerotic lesions upon intraplaque hemorrhage [10]. When exposed to Hb, oxidized Hb, or heme, HASMCs and macrophages upregulated the expression of CSE, whereas iron treatment caused a marked decrease of CSE expression in HASMCs, but did not modulate CSE expression in macrophages (Figure 8(b), left and middle panels). We also examined the upregulation of CSE by various forms of hemoglobin, lipids, and proinflammatory cytokines in HAoECs. CSE expression was not altered in HAoECs exposed to oxHb, oxLDL, and oxidized lipids derived from atheromatous plaque (Figures 8(a) and 8(b), right panels). To examine the effect of proinflammatory mediators, we next exposed the cells to IL-1*β* and TNF-*α*. Figures 8(c) shows that CSE expression was induced in HASMCs and macrophages after cytokine stimulation. On the contrary, CSE level was significantly increased in cells treated with TNF-*α* as it was observed in HAoECs (Figure 8(c), right panels).

## 4. Discussion

The oxidative environment in the atherosclerotic lesion triggers lysis of invading erythrocytes and the subsequent release of Hb upon intraplaque hemorrhage [10]. A complex interplay exists between atheroma lipids and cell-free Hb that leads to further lipid-peroxidation and Hb oxidation [10–12, 22].

In the reactions between Hb and plaque lipids, different oxidized Hb derivatives are formed (metHb (Fe^3+^), ferrylHb (Fe^4+^=O^2−^)) [10–12], resulting in the covalently cross-linked species. In our current study, we demonstrated that sulfide inhibited oxidative cross-linking of Hb subunits, the hallmark of the ferrylHb formation. These effects could be attributed to the ability of sulfide to reduce LOOH content of oxLDL because LOOH was identified previously as the most cytotoxic lipid-peroxidation product in oxLDL toward vascular endothelium [22]. However, as we argued previously, the antioxidant properties of sulfide in biological systems are most likely not due to direct scavenging of oxidant species, because protein thiols and GSH are present in much larger concentrations compared to sulfide. Hb cross-linking proceeds via ferrylHb intermediate species, those we found to be scavenged rapidly by sulfide, hence providing an alternative molecular model for sulfide-mediated inhibition of Hb oligomerization. The measured rate constants for the reactions of ferrylHb species with sulfide represent at least 1–3 orders of magnitude faster reactions (for the slower reactions recorded at 425 nm and the faster ones at 620 nm, resp.) compared to those with ascorbate or urate [28] that are the previously proposed primary heme-redox-intermediate-scavenging antioxidants (because thiols in most cases have no access to the metal center at the active site). Also, the relatively high stability of the ferrylHb species implies that Hb oligomers must form in much slower reactions. Furthermore, the fact that Hb oligomerization was associated with intraplaque hemorrhage suggests a role for ferrylHb-initiated oxidative stress in the underlying molecular mechanisms of the pathophysiology of complicated atherosclerotic lesions [10]. Hence, the demonstration of sulfide-mediated reduction of ferrylHb species is a novel observation which could explain at least some of the observed cytoprotective effects of sulfide in our current model and adds to the rapidly growing body of literature detailing H_2_S-mediated cytoprotection.

Additionally, oxidized Hb species, in particular ferrylHb, induce proinflammatory signaling targeted to the vascular endothelium. By inhibiting Hb oxidation, sulfide donors also attenuated Hb oxidation-associated decrease in endothelial monolayer integrity and intercellular gap formation, as well as the induction of the adhesion molecule, VCAM-1.

Oxidized Hb species release heme, a potent trigger of lipid peroxidation [14, 25]. In our current study, sulfide donors inhibited Hb or heme-mediated oxidation of soft plaque lipids derived from human atheroma. We have also shown that sulfide-releasing molecules attenuated lipid peroxidation in complicated lesions exhibiting evidence of hemorrhage. Chemically, H_2_S is a reductant, and the previous study revealed that it converts lipid hydroperoxides into lipid-alcohols in oxLDL *in vitro* [29]. Importantly, we found that sulfide reduced lipid hydroperoxide content of complicated lesions with hemorrhage.

Using human carotid artery specimens and mice aorta derived from an experimental atherosclerosis model, here, we demonstrated that the expression of CSE is markedly upregulated in vessels with atheromatous plaques and complicated lesions. Macrophages, activated myofibroblasts, and foam cells exhibited marked CSE expression in the lesions, and interestingly, the CSE-positive cells were more common in atheromatous plaques compared to complicated lesions. The atherosclerotic plaque is rich in reactive oxygen species, reactive lipid mediators, proinflammatory cytokines, and upon plaque hemorrhage, contains different hemoglobin oxidation products. Here, we showed that all the major cell types of the atherosclerotic vessel wall, that is, endothelial cells, vascular smooth muscle cells, and macrophages, expressed CSE in a stress-responsive manner. We identified proinflammatory cytokines (TNF-*α*, IL-1*β*), lipid mediators (oxLDL, plaque lipid), and hemoglobin oxidation products (ferrylHb) that could contribute the high expression of CSE in blood vessels obtained from human atheromatous plaques and complicated lesions.

As we investigated the effects of a CSE inhibitor (PPG) in atherosclerotic plaque formation *in vivo*, we found that PPG treatment increased plaque formation in the presence of an atherogenic diet confirming Wang et al.'s and Mani et al.'s previous findings [17, 18]. This suggests that the CSE/H_2_S system inhibits the development of atheromatous lesions. Unfortunately, in ApoE^−/−^ mice model, there is no evidence of the development of complicated lesions. To our knowledge, there is no animal model for studying the fate of complicated lesions. In human, the main pathogenic event in the progression of atherosclerosis is the formation of complicated lesions with obvious macroscopic evidence of intraplaque hemorrhage. We demonstrate that complicated lesions with obvious macroscopic evidence of intraplaque hemorrhage in humans exhibit less CSE expression compared to an atheromatous plaque. These data support the beneficial role of CSE/H_2_S in the progression of atherosclerosis and does not provide evidence for an association of CSE upregulation and atherosclerosis. It should be noted that there is no animal model available at the moment for verifying the function of H_2_S for controlling the prooxidant and proinflammatory ferrylHb. Our aim is in the future to establish a model for the development and progression of complicated lesions. Prevention of plaque rupture and infiltration of red blood cells into the atheroma might provide a novel approach to inhibit the progression of lesions in human.

**Figure 9:**
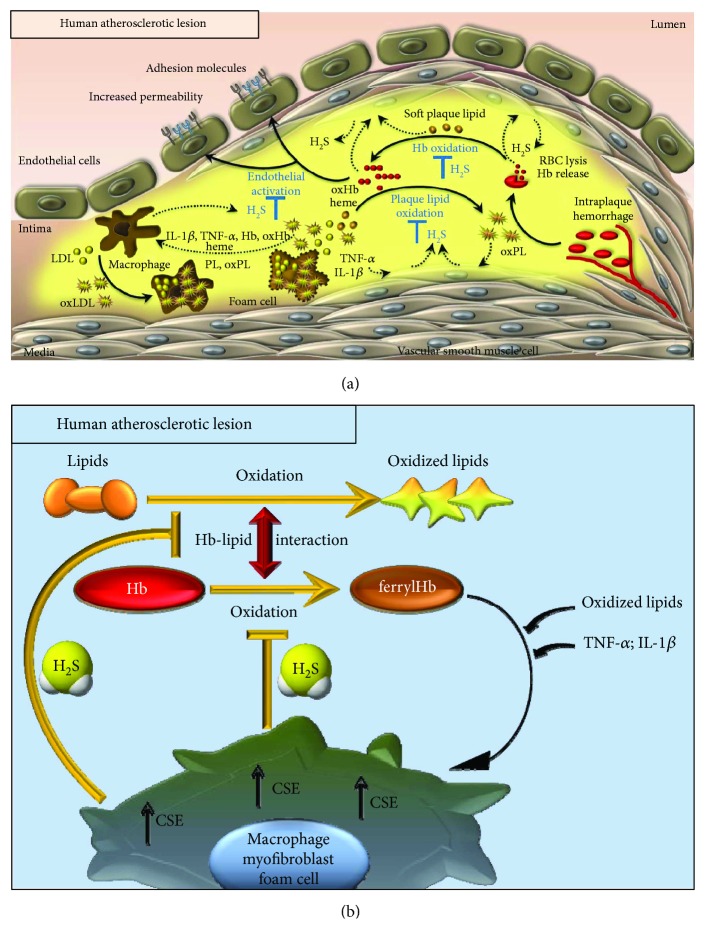
Proposed protective actions of H_2_S in the atherosclerotic plaque. (a) Upon infiltration of RBCs into the atherosclerotic lesion erythrocytes are lysed and oxidation of liberated Hb occurs followed by heme release. Oxidized Hb forms and the released heme trigger further lipid peroxidation. FerrylHb exhibits proinflammatory property provoking endothelial cell activation characterized by intercellular gap formation and increased adhesion molecule expression. Endothelial activation facilitates monocyte adhesion and transendothelial migration. (b) In the reactions between Hb and plaque lipids, different oxidized Hb derivatives are formed including metHb and ferrylHb species. Macrophages, foam cells, and smooth muscle cell-derived myofibroblasts respond to such an insult (ferrylHb, heme, plaque lipids, and the proinflammatory cytokines IL-1*β* and TNF-*α*) by upregulating CSE expression. The increased production of H_2_S inhibits (i) oxidation of Hb preventing the formation of ferrylHb derivatives (a novel function as a heme-redox-intermediate-scavenging antioxidant), (ii) oxidation of plaque lipids, and subsequently (iii) activation of endothelium.

In conclusion, the present study provides evidence that high vascular expression of CSE defines atheromatous plaques and complicated lesions, both in human carotid atherosclerotic lesions and in an experimental hyperlipidemia-induced atherosclerotic mouse model. Based on our *in vitro* findings, we propose that the elevated CSE expression might serve as a compensatory atheroprotective response, in which the produced H_2_S prevents the formation of prooxidant and proinflammatory lipid mediators and Hb forms and subsequent endothelial responses provoked by these species (Figures 9(a) and 9(b)).

## Supplementary Materials

Supplementary Materials Supplementary Figure 1: CSE is markedly upregulated in human atheromatous lesions immunohistochemistry detection of the CSE expression in human carotid artery specimens. H&E and CSE staining are shown. Magnification of the human histology samples was 150x. Arrows indicate endothelial cells, crosses show smooth muscle cells, and comparison signs mark macrophages. Supplementary Figure 2: CSE is markedly upregulated in mouse atheromatous lesions. ApoE^−/−^ mice were kept on normal chow diet or atherogenic diet for 8 weeks. H&E and CSE staining are shown. Magnification of the mice aortas was 700x. Arrows indicate endothelial cells, crosses show smooth muscle cells, and comparison signs mark macrophages. Supplementary Figure 3: sulfide donors were inhibited lipid peroxidation *in vitro.* (A-B) Soft plaque lipids (400 *μ*g/mL) were incubated with heme (5 *μ*mol/L) alone or in the presence of sulfide donors NaSH, GYY4137, AP67, and AP72 (200 *μ*mol/L) at 37°C for 24 hours, followed by determination of TBARS and LOOH. Representative experiment, *N* = 3, each performed in triplicate. (C-D) Soft plaque lipids (400 *μ*g/mL) were incubated with Hb (20 *μ*mol/L) alone or in the presence of sulfide donors NaSH, GYY4137, AP67, and AP72 (200 *μ*mol/L) at 37°C for 48 hours, followed by determination of TBARS and LOOH. Representative experiment, *n* = 3, each performed in triplicate. Supplementary Figure 4: sulfide inhibits lipid-peroxidation in complicated lesions. (A) Macroscopic picture, H&E staining, and Hb staining of human complicated lesions are shown. (B-C) Human carotid artery specimens with obvious macroscopic evidence of intraplaque hemorrhage were homogenized. The homogenate (5 mg/mL) was incubated in the presence or absence of the sulfide donors NaSH, GYY4137, AP67, and AP72 (200 *μ*mol/L) at 37°C for 4 days. LOOH and TBARS levels were determined on day 0 and day 4. Representative experiment, *n* = 3. Supplementary Figure 5: polysulfides and decomposed sulfide donors not able to block the lipid peroxidation. (A) LDL (200 *μ*g/ml) was incubated with heme (5 *μ*mol/L) and H_2_O_2_ in the presence or absence of HSX^−^ or decomposed sulfide donors NaSH, GYY4137, AP67, and AP72 at the concentrations of 20 and 200 *μ*mol/L at 37°C for 60 minutes followed by determination of TBARS and LOOH. Representative experiment, *n* = 3, each performed in triplicate. (B) Soft plaque lipids (400 *μ*g/mL) were incubated with heme (5 *μ*mol/L) alone or in the presence of HSX^−^ or decomposed sulfide donors NaSH, GYY4137, AP67, and AP72 (200 *μ*mol/L) at 37°C for 16 hours, followed by determination of TBARS and LOOH. Representative experiment, *n* = 3, each performed in triplicate. (C) Soft plaque lipids (400 *μ*g/mL) were incubated with Hb (20 *μ*mol/L) alone or in the presence of HSX^−^ or decomposed sulfide donors NaSH, GYY4137, AP67, and AP72 (200 *μ*mol/L) at 37°C for 16 hours, followed by determination of TBARS and LOOH. Representative experiment, *n* = 3, each performed in triplicate. Supplementary Figure 6: polysulfides and decomposed sulfide donors do not prevent Hb-lipid interactions on endothelial cells. (A-B) OxLDL (200 *μ*g/mL) was incubated with HSX^−^ or decomposed sulfide donors NaSH, GYY4137, AP67, and AP72 at the concentration of 200 *μ*mol/L at 37°C for 16 hours. HUVECs were treated with the samples for 6 hours then cell viability was determined by MTT assay. Representative experiment, *n* = 3, each performed in 8 wells in parallel. OxLDL (200 *μ*g/mL) was incubated with HSX^−^ or decomposed sulfide donors NaSH, GYY4137, AP67, and AP72 at the concentration of 200 *μ*mol/L at 37°C for 16 hours. (C) HUVECs were treated to LDL samples (50 *μ*g/mL) for 8 hours. HO-1 protein expression was determined by Western blotting. Representative experiment, *n* = 3. Relative densitometry was determined with ImageJ software. (D) Hb (10 *μ*mol/L) was incubated with H_2_O_2_ (50 *μ*mol/L) in the presence or absence of HSX^−^ or decomposed sulfide donors NaSH, GYY4137, AP67, and AP72 (200 *μ*mol/L) at 37°C for 90 minutes. HUVECs were exposed to the obtained Hb samples for 8 hours and VCAM-1 expression was determined by Western blotting. Representative experiment, *n* = 3. Relative densitometry was determined with ImageJ software.
