# Supplementary material for: Hydrogen Sulfide Abrogates Hemoglobin-Lipid Interaction in Atherosclerotic Lesion
Source: Oxid Med Cell Longev. 2018 Jan 21;2018:3812568. doi: 10.1155/2018/3812568 (PMC5828047; doi:10.1155/2018/3812568)
Supplement: Supplementary Materials — Supplementary Figure 1: CSE is markedly upregulated in human atheromatous lesions immunohistochemistry detection of the CSE expression in human carotid artery specimens. H&E and CSE staining are shown. Magnification of the human histology samples was 150x. Arrows indicate endothelial cells, crosses show smooth muscle cells, and comparison signs mark macrophages. Supplementary Figure 2: CSE is markedly upregulated in mouse atheromatous lesions. ApoE−/− mice were kept on normal chow diet or atherogenic diet for 8 weeks. H&E and CSE staining are shown. Magnification of the mice aortas was 700x. Arrows indicate endothelial cells, crosses show smooth muscle cells, and comparison signs mark macrophages. Supplementary Figure 3: sulfide donors were inhibited lipid peroxidation in vitro. (A-B) Soft plaque lipids (400 μg/mL) were incubated with heme (5 μmol/L) alone or in the presence of sulfide donors NaSH, GYY4137, AP67, and AP72 (200 μmol/L) at 37°C for 24 hours, followed by determination of TBARS and LOOH. Representative experiment, N = 3, each performed in triplicate. (C-D) Soft plaque lipids (400 μg/mL) were incubated with Hb (20 μmol/L) alone or in the presence of sulfide donors NaSH, GYY4137, AP67, and AP72 (200 μmol/L) at 37°C for 48 hours, followed by determination of TBARS and LOOH. Representative experiment, n = 3, each performed in triplicate. Supplementary Figure 4: sulfide inhibits lipid-peroxidation in complicated lesions. (A) Macroscopic picture, H&E staining, and Hb staining of human complicated lesions are shown. (B-C) Human carotid artery specimens with obvious macroscopic evidence of intraplaque hemorrhage were homogenized. The homogenate (5 mg/mL) was incubated in the presence or absence of the sulfide donors NaSH, GYY4137, AP67, and AP72 (200 μmol/L) at 37°C for 4 days. LOOH and TBARS levels were determined on day 0 and day 4. Representative experiment, n = 3. Supplementary Figure 5: polysulfides and decomposed sulfide donors not able to block the lipid [file 3812568.f1.pdf]

Supplementary Figure 1.

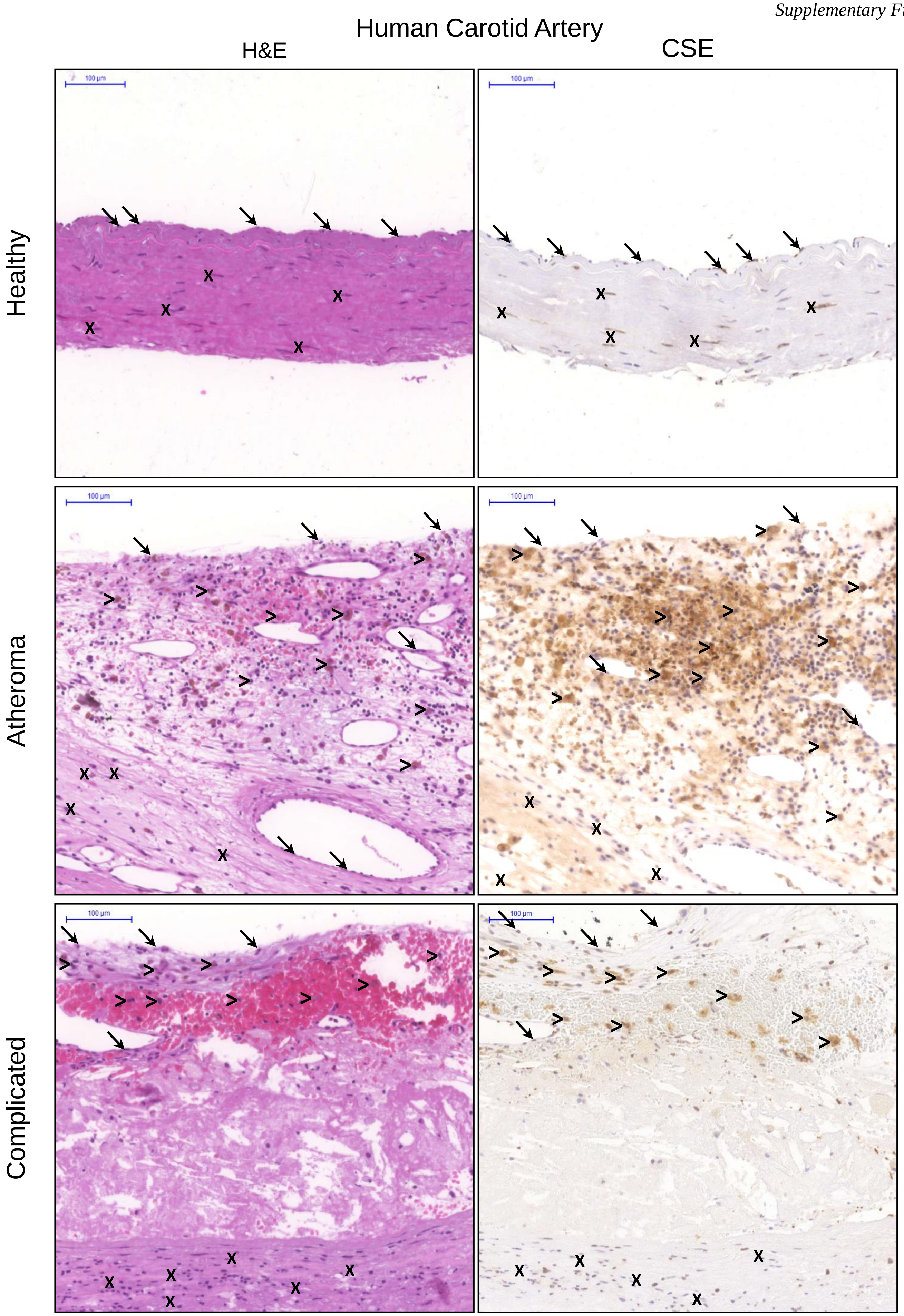

Supplementary Figure 2.

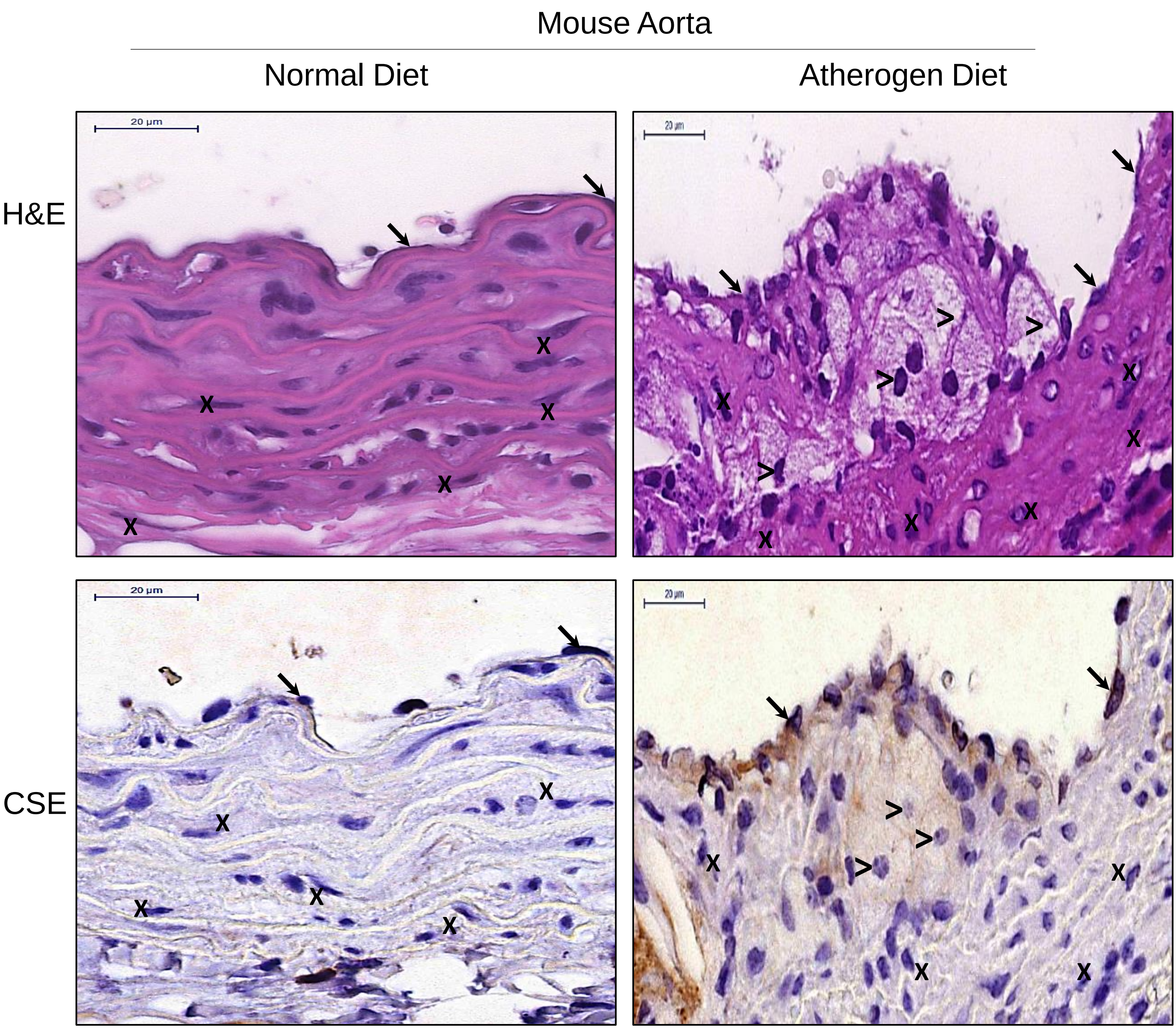

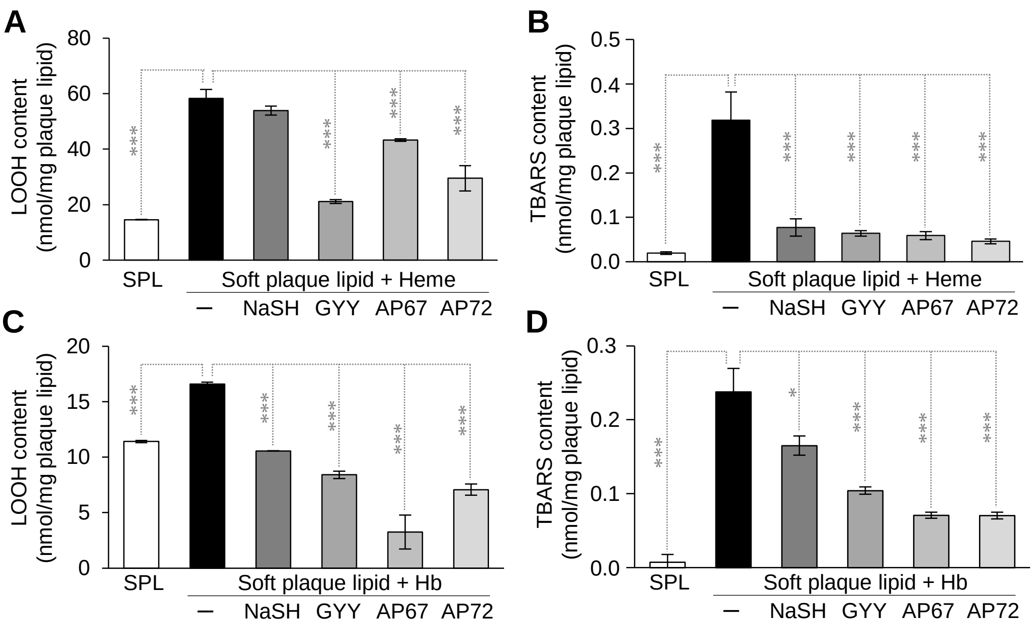
A
LOOH content
(nmol/mg plaque lipid)
80
60
40
20
0
SPL
Soft plaque lipid + Heme
—
NaSH
GYY
AP67
AP72
B
TBARS content
(nmol/mg plaque lipid)
0.5
0.4
0.3
0.2
0.1
0.0
SPL
Soft plaque lipid + Heme
—
NaSH
GYY
AP67
AP72
C
LOOH content
(nmol/mg plaque lipid)
20
15
10
5
0
SPL
Soft plaque lipid + Hb
—
NaSH
GYY
AP67
AP72
D
TBARS content
(nmol/mg plaque lipid)
0.3
0.2
0.1
0.0
SPL
Soft plaque lipid + Hb
—
NaSH
GYY
AP67
AP72

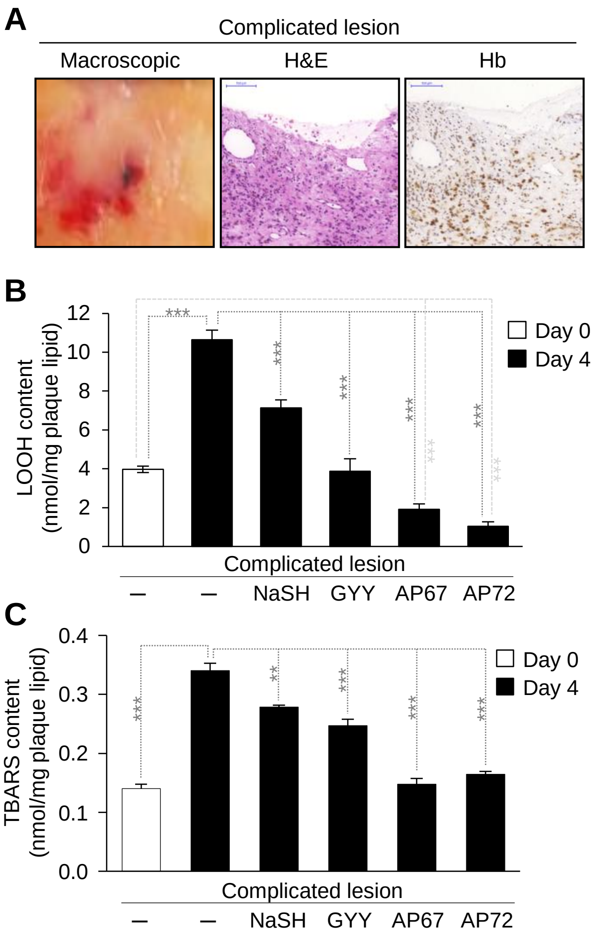
A
Complicated lesion
Macroscopic
H&E
Hb
B
LOOH content
(nmol/mg plaque lipid)
12
10
8
6
4
2
0
***
Day 0
Day 4
Complicated lesion
—
—
NaSH
GYY
AP67
AP72
C
TBARS content
(nmol/mg plaque lipid)
0.4
0.3
0.2
0.1
0.0
***
**
Day 0
Day 4
Complicated lesion
—
—
NaSH
GYY
AP67
AP72

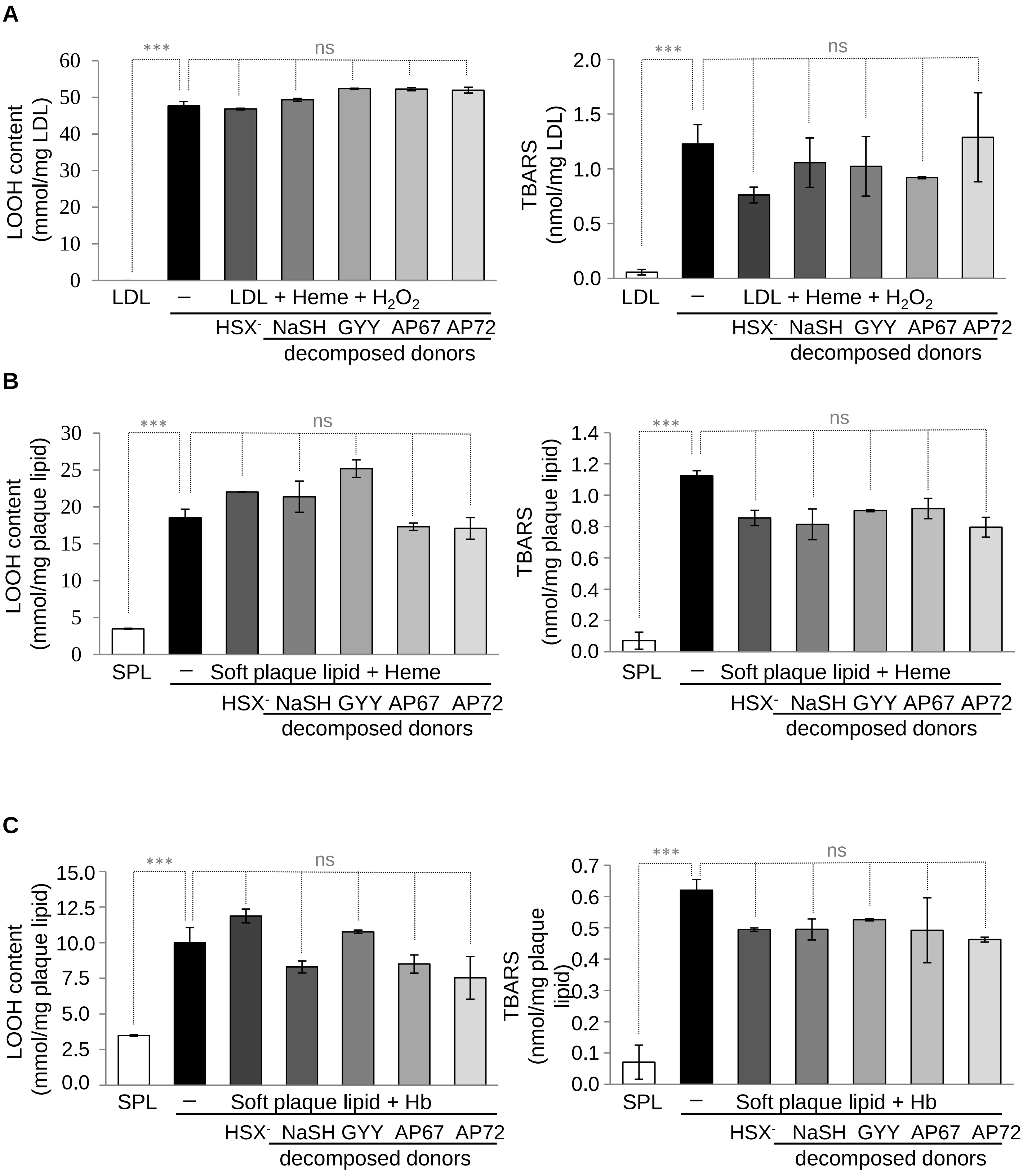
A
***
ns
LOOH content (mmol/mg LDL)
0
10
20
30
40
50
60
LDL
–
LDL + Heme + H2O2
HSX-
NaSH
GYY
AP67
AP72
decomposed donors
TBARS (nmol/mg LDL)
0.0
0.5
1.0
1.5
2.0
B
LOOH content (mmol/mg plaque lipid)
0
5
10
15
20
25
30
SPL
Soft plaque lipid + Heme
TBARS (nmol/mg plaque lipid)
0.0
0.2
0.4
0.6
0.8
1.0
1.2
1.4
C
LOOH content (mmol/mg plaque lipid)
0.0
2.5
5.0
7.5
10.0
12.5
15.0
Soft plaque lipid + Hb
TBARS (nmol/mg plaque lipid)
0.0
0.1
0.2
0.3
0.4
0.5
0.6
0.7

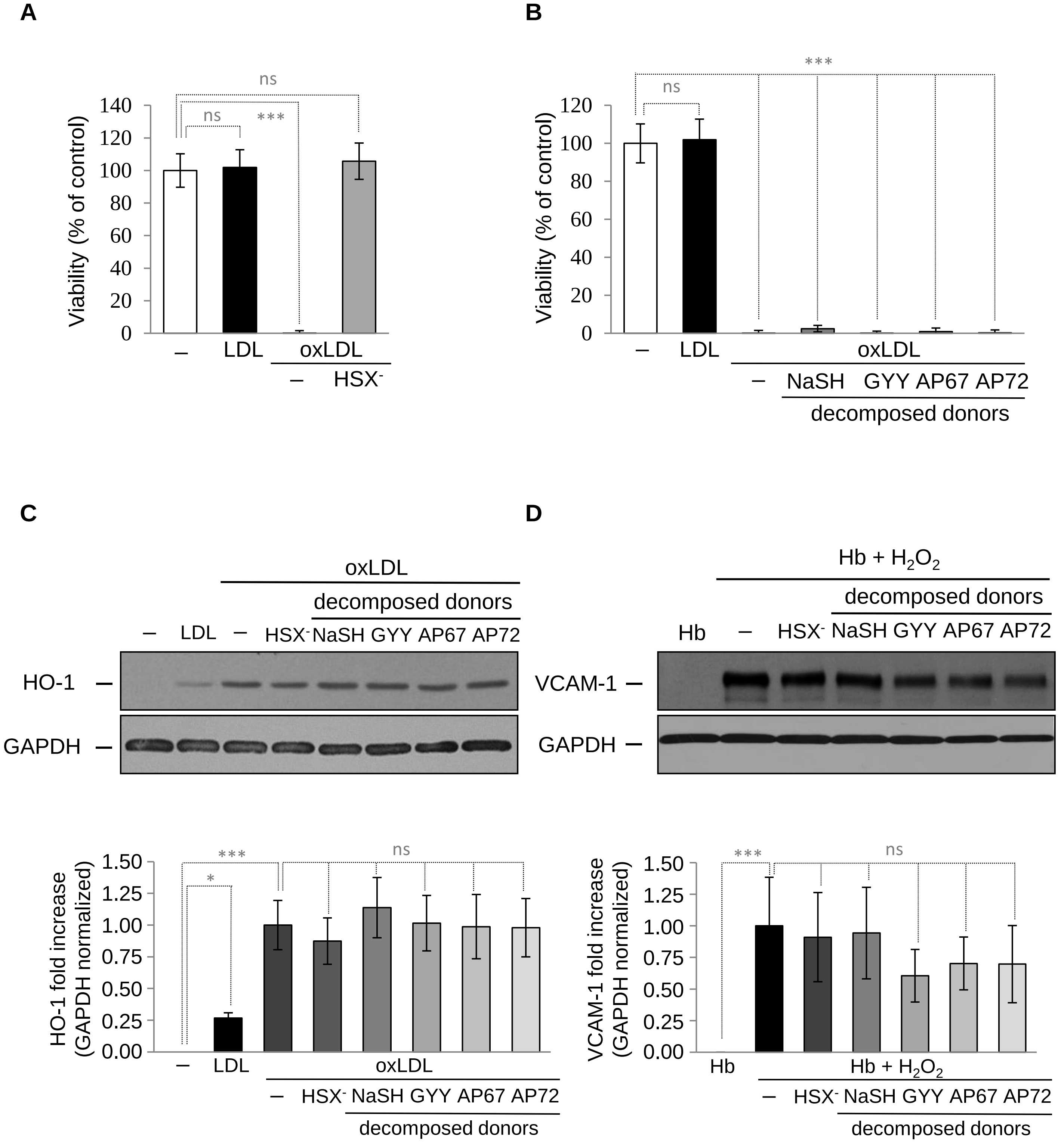
A
ns
ns
***
Viability (% of control)
140
120
100
80
60
40
20
0
–
LDL
oxLDL
–
HSX-
B
***
ns
Viability (% of control)
120
100
80
60
40
20
0
–
LDL
oxLDL
–
NaSH
GYY
AP67
AP72
decomposed donors
C
oxLDL
decomposed donors
– LDL – HSX- NaSH GYY AP67 AP72
HO-1
GAPDH
D
Hb + H2O2
decomposed donors
Hb – HSX- NaSH GYY AP67 AP72
VCAM-1
GAPDH
***
*
ns
HO-1 fold increase (GAPDH normalized)
1.50
1.25
1.00
0.75
0.50
0.25
0.00
– LDL
oxLDL
– HSX- NaSH GYY AP67 AP72
decomposed donors
***
ns
VCAM-1 fold increase (GAPDH normalized)
1.50
1.25
1.00
0.75
0.50
0.25
0.00
Hb
Hb + H2O2
– HSX- NaSH GYY AP67 AP72
decomposed donors

SUPPLEMENTARY FIGURE LEGENDS

Supplementary Figure 1. CSE is markedly upregulated in human atheromatous lesions

Immunohistochemistry detection of the CSE expression in human carotid artery specimens. H&E and CSE staining, are shown. Magnification of the human histology samples was 150x. Arrows indicate endothelial cells; crosses show smooth muscle cells and comparison signs mark macrophages.

Supplementary Figure 2. CSE is markedly upregulated in mouse atheromatous lesions

$ApoE^{-/-}$ mice were kept on normal chow diet or atherogenic diet for 8 weeks. H&E and CSE staining are shown. Magnification of the mice aortas was 700x. Arrows indicate endothelial cells; crosses show smooth muscle cells and comparison signs mark macrophages.

Supplementary Figure 3. Sulfide donors were inhibited lipid peroxidation *in vitro*

(A-B) Soft plaque lipids (400 µg/mL) were incubated with heme (5 µmol/L) alone or in the presence of sulfide donors NaSH, GYY4137, AP67 and AP72 (200 µmol/L) at 37°C for 24 hours, followed by determination of TBARS and LOOH. Representative experiment, n=3, each performed in triplicate. (C-D) Soft plaque lipids (400 µg/mL) were incubated with Hb (20 µmol/L) alone or in the presence of sulfide donors NaSH, GYY4137, AP67 and AP72 (200 µmol/L) at 37°C for 48 hours, followed by determination of TBARS and LOOH. Representative experiment, n=3, each performed in triplicate.

Supplementary Figure 4. Sulfide inhibits lipid-peroxidation in complicated lesions

(A) Macroscopic picture, H&E staining and Hb staining of human complicated lesions are shown. (B-C) Human carotid artery specimens with obvious macroscopic evidence of intraplaque hemorrhage were homogenized. The homogenate (5 mg/mL) was incubated in the presence or absence of the sulfide donors NaSH, GYY4137, AP67 and AP72 (200

μmol/L) at 37°C for 4 days. LOOH and TBARS levels were determined on day 0 and day 4. Representative experiment, n=3.

Supplementary Figure 5. Polysulfides and decomposed sulfide donors not able to block the lipid peroxidation

(A) LDL (200 μg/ml) was incubated with heme (5 μmol/L) and H_2O_2 in the presence or absence of HSX^- or decomposed sulfide donors NaSH, GYY4137, AP67 and AP72 at the concentrations of 20 and 200 μmol/L at 37°C for 60 minutes followed by determination of TBARS and LOOH. Representative experiment, n=3, each performed in triplicate. (B) Soft plaque lipids (400 μg/mL) were incubated with heme (5 μmol/L) alone or in the presence of HSX^- or decomposed sulfide donors NaSH, GYY4137, AP67 and AP72 (200 μmol/L) at 37°C for 16 hours, followed by determination of TBARS and LOOH. Representative experiment, n=3, each performed in triplicate. (C) Soft plaque lipids (400 μg/mL) were incubated with Hb (20 μmol/L) alone or in the presence of HSX^- or decomposed sulfide donors NaSH, GYY4137, AP67 and AP72 (200 μmol/L) at 37°C for 16 hours, followed by determination of TBARS and LOOH. Representative experiment, n=3, each performed in triplicate.

Supplementary Figure 6. Polysulfides and decomposed sulfide donors do not prevent Hb-lipid interactions on endothelial cells

(A-B) OxLDL (200 μg/mL) was incubated with HSX^- or decomposed sulfide donors NaSH, GYY4137, AP67 and AP72 at the concentration of 200 μmol/L at 37°C for 16 hours. HUVECs were treated with the samples for 6 hours then cell viability was determined by MTT assay. Representative experiment, n=3, each performed in 8 wells in parallel. OxLDL (200 μg/ml) was incubated with HSX^- or decomposed sulfide donors NaSH, GYY4137, AP67 and AP72 at the concentration of 200 μmol/L at 37°C for 16 hours. (C) HUVECs were treated to LDL samples (50 μg/mL) for 8 hours. HO-1 protein expression was determined by Western

blotting. Representative experiment, n=3. Relative densitometry was determined with ImageJ software. (D) Hb (10 μmol/L) was incubated with H_2O_2 (50 μmol/L) in the presence or absence of HSX^- or decomposed sulfide donors NaSH, GYY4137, AP67 and AP72 (200 μmol/L) at 37°C for 90 minutes. HUVECs were exposed to the obtained Hb samples for 8 hours and VCAM-1 expression was determined by Western blotting. Representative experiment, n=3. Relative densitometry was determined with ImageJ software.

SUPPLEMENTARY MATERIALS AND METHODS

Materials

All chemicals were analytical reagent grade or better and purchased from Sigma-Aldrich, (St Louis, MO, USA). The sulfide donor molecules used in this study − GYY4137, AP67 and AP72 − were synthesized in-house [1, 2]. Sulfide stock solutions were prepared fresh daily in water and used immediately.

Tissue samples

Carotid artery plaques were collected from patients underwent carotid endarterectomy surgery.

Immunohistochemistry

Immunohistochemistry from the carotical arteries was performed on formalin-fixed, paraffin-embedded tissue sections. 4 μm slides were then deparaffinated using xylol and ethanol.

Samples were incubated with the anti-CSE primary monoclonal antibody (12217-1-AP Proteintech, Chicago, IL, USA) at a dilution of 1:200. Other slides of the same samples were incubated with anti-hemoglobin (clone: goat polyclonal HRP - ab19362 Proteintech Group, Rosemont, IL 60018, USA) primary monoclonal antibody at a dilution of 1:100; with HO-1 (clone: rabbit polyclonal 10701-1-AP Proteintech Group, Rosemont, IL 60018, USA) primary monoclonal antibody at a dilution of 1:200; and with HO-2 (clone: rabbit polyclonal 14817-1-AP Proteintech Group, Rosemont, IL 60018, USA) primary monoclonal antibody at a dilution of 1:400. Specific antibody binding was visualized by the Dako EnVision FLEX/HRP and FLEX DAB3 Chromogen detection system (Dako, Glostrup, Danmark) followed by hematoxylin counterstaining and coverage. The intensity and distribution of proteins immunostaining were assessed by light microscopy (Leica DM2500 microscope, DFC 420 camera and Leica Application Suite V3 software, Leica).

Preparation of polysulfide solutions

Polysulfides were prepared by mixing 2 mmol/L Sodium hypochlorite (NaOCl) and 10 mmol/L Sodium sulfide (Na_2S). NaOCl was adding dropwise to the Hs^- solution with a continuous vortex. All the procedures were performed on ice and in the dark. Polysulfide was used within 30 minutes. Concentrated NaOCl was diluted in 50 mmol/L PBS (pH 7.4). Absorbance was determined by spectrophotometer at 292 nm and the concentration was calculated with extinction coefficient (ex.coeff: 350 cm^{-1} M^{-1}).

Preparation of decomposed sulfide donors

We dissolved sulfide donors in PBS and let them release their entire sulfide. We continuously monitored the sulfide content by spectrophotometer at 620 nm. We had to wait over one month to full decomposition of the slow releasing molecules.

Hemoglobin preparation

Hb of different redox states, i.e. (Fe^{2+}) oxyHb, (Fe^{3+}) metHb, and ferrylHb, were prepared as described [3]. Briefly, Hb was isolated from fresh blood drawn from healthy volunteers using ion-exchange chromatography on a DEAE Sepharose CL-6B column. MetHb was generated by incubation (30 min, 25°C) of purified Hb with a 1.5-fold molar excess of $K_3Fe(CN)_6$ over heme. FerrylHb was obtained by incubation (1 h, 37°C) of Hb with a 10:1 ratio of H_2O_2 to heme. After oxidation, both metHb and ferrylHb were dialyzed against saline (3 times for 3 hours at 4°C) and concentrated using Amicon Ultra centrifugal filter tubes (10,000 MWCO, Millipore Corp., Billerica, MA, USA). Aliquots were snap-frozen in liquid nitrogen, and stored at -80°C until use. The purity of each Hb preparation was evaluated by SDS-PAGE followed by silver staining. The purity of Hb preparations was above 99.9%. Hb concentrations were calculated as described by Winterbourn [4].

Isolation and oxidation of LDL

LDL was isolated from the plasma of EDTA-anticoagulated venous blood of healthy volunteers by gradient ultracentrifugation (Beckman Coulter Inc., Brea, CA, USA). The density of plasma was adjusted to 1.3 g/mL with KBr and a two-layer gradient was made in a Quick-Seal ultracentrifuge tube by layering saline on 10 ml plasma. Ultracentrifugation was performed at 302,000 g for 2 hours at 4°C (VTi 50.2 rotor). LDL samples were kept at -70°C until use and the protein concentration was determined by Pierce BCA protein assay Kit (Pierce Biotechnology, Rockford, IL, USA). LDL oxidation was carried out at 37°C in a reaction mixture containing LDL (200 μg/mL) heme (5 μmol/L) and H_2O_2 (75 μmol/L).

Oxidation of LDL

LDL (200 μg/mL) was oxidized with heme (5 μmol/L) and H_2O_2 (75 μmol/L) in the presence or absence of the sulfide donors NaSH, GYY4137, AP67 and AP72 at concentrations of 20 and 200 μmol/L at 37 °C. Conjugated diene formation was monitored continuously for 1 hour at 234 nm. Delta OD234 nm was calculated by subtracting optical density measured at a 0-time point from optical density measured at 1 hour. The formations of lipid hydroperoxides (LOOH) and thiobarbituric-acid reactive substances (TBARS) were measured at 60 minutes following the initiation of lipid peroxidation. The method of Wolf was used to evaluate LOOH content in the LDL samples [5]. For TBARS measurement, 50 μL of a 200 μg protein/ml LDL sample was mixed with 100 μL of thiobarbituric acid reagent (0.375 g 2-thiobarbituric acid, 2.08 mL HCl, 15 mL 10% trichloroacetic acid to a final volume of 100 mL). After heating at 90°C for 20 minutes, the samples were cooled and extracted with 200 μL n-butanol. The upper phase was measured spectrophotometrically at 532 nm. Results were calculated using a molar extinction coefficient of $1.56 \times 10^5\ M^{-1}cm^{-1}$ and are expressed as nmol TBARs/mg protein.

Plaque lipid oxidation

Lipids were extracted from human carotid artery plaques as described previously (2). Plaque lipids (0.5 mg/mL) were incubated with Hb (100 μmol/L) in the presence or absence of the sulfide donors NaSH, GYY4137, AP67 and AP72 at 200 μmol/L concentration for 4 days at 37 °C. In other cases complicated lesions containing intraplaque hemorrhage were homogenized in saline. These samples (0.5 mg/mL) were incubated at 37 °C for 3 days in the presence or absence of the sulfide donors NaSH, GYY4137, AP67 and AP72. Lipid peroxidation was assessed by measuring LOOH and TBARs

Endothelial cell cytotoxicity assay

LDL (200 μg/mL) was oxidized with heme (5 μmol/L) and H_2O_2 (75 μmol/L). Oxidized LDL was incubated at 37°C overnight with the sulfide donors NaSH, GYY4137, AP67 and AP72 at concentrations of 20 and 200 μmol/L. Confluent endothelial cells grown in 96-well tissue-culture plates were washed twice with PBS and exposed to oxLDL samples for 6 hours. Cell viability was assessed by MTT assay as described previously [6].

Western blot

Cells were cultured in 6-well plates and treated with different triggers. After 8 hours of treatment the cells were solubilized in protein lysis buffer containing 10 mmol/L Tris-HCl, 5 mmol/L EDTA, 150 mmol/L NaCl (pH 7.2), 1% Triton X-100, 0.5% Nonidet P-40 and protease inhibitors (Complete Mini, F. Hoffmann-La Roche Ltd., Basel, Switzerland). In other experiments, tissue samples were homogenized under liquid nitrogen and solubilized in protein lysis buffer. Proteins (10-20 μg) were applied to 12.5% SDS-PAGE gels. After electrophoresis, proteins were transferred to a nitrocellulose membrane (Amersham Biosciences Corp., Piscataway, NJ, USA). Proteins were identified using the following antibodies: mouse anti-human HO-1 antibody (Calbiochem, San Diego, CA, USA, 374087, dilution: 1:2500), rabbit anti-human CSE antibody (Proteintech, Chicago, IL, USA, 12217-1-AP, dilution: 1:1000), rabbit anti-human VCAM-1 (Santa Cruz Biotechnology Inc., Dallas,

TX, USA, sc8304, dilution: 1:200), mouse anti-human GAPDH (Novus Biologicals, Littleton, CO, USA NB-300-221, dilution: 1:1000), anti-rabbit IgG HRP-conjugated (GE Healthcare Life Sciences, Piscataway, NJ, USA, NA934, dilution 1:15000), and anti-mouse IgG HRP-conjugated (GE Healthcare Life Sciences, Piscataway, NJ, USA, NA931, dilution 1:15000). Antigen-antibody complex was detected by a horseradish peroxidase chemiluminescence system according to the manufacturer's instructions (GE Healthcare Life Sciences, Piscataway, NJ, USA,). Quantification was performed using video densitometry (AlphaDigiDoc RT, Alpha Innotech Corp., San Leandro, CA, USA).

Experimental units

"N" represents the number of tissue samples used in each group. The "n" denoting the number of replications of the independent results.

Statistics

Data were analyzed by GraphPad Prism 5.02 software (GraphPad Software, Inc. 7825 Fay Avenue, Suite 230 La Jolla, CA 92037). All statistics data are expressed as mean ± s.e.m. Differences in means were analyzed by Student's t-test or one-way ANOVA with Dunnett post test as appropriate. $P<0.05$ was considered significant.

Study approval

A collection of carotid artery plaques from patients underwent carotid endarterectomy surgery was approved by the Scientific and Research Ethics Committee of the Scientific Council of Health of the Hungarian Government under the registration number of DE OEC RKEB/IKEB 3712-2012.

REFERENCES

1. Chitnis MK, Njie-Mbye YF, Opere CA, *et al.* Pharmacological actions of the slow release hydrogen sulfide donor GYY4137 on phenylephrine-induced tone in isolated bovine ciliary artery. *Exp Eye Res* 2013; **116**: 350-354.

2. Whiteman M, Li L, Rose P, *et al.* The effect of hydrogen sulfide donors on lipopolysaccharide-induced formation of inflammatory mediators in macrophages. *ANTIOXIDANTS & REDOX SIGNALING* 2010; **12**: 1147-1154.

3. Silva G, Jeney V, Chora A, *et al.* Oxidized hemoglobin is an endogenous proinflammatory agonist that targets vascular endothelial cells. *The Journal of biological chemistry* 2009; **284**: 29582-29595.

4. Winterbourn CC. Oxidative reactions of hemoglobin. *Methods in enzymology* 1990; **186**: 265-272.

5. Jeney V, Balla J, Yachie A, *et al.* Pro-oxidant and cytotoxic effects of circulating heme. *Blood* 2002; **100**: 879-887.

6. Nagy E, Jeney V, Yachie A, *et al.* Oxidation of hemoglobin by lipid hydroperoxide associated with low-density lipoprotein (LDL) and increased cytotoxic effect by LDL oxidation in heme oxygenase-1 (HO-1) deficiency. *Cell Mol Biol (Noisy-le-grand)* 2005; **51**: 377-385.
